# Effectiveness of Internet-Based Exercises Aimed at Treating Knee Osteoarthritis: The iBEAT-OA Randomized Clinical Trial

**DOI:** 10.1001/jamanetworkopen.2021.0012

**Published:** 2021-02-23

**Authors:** Sameer Akram Gohir, Frida Eek, Anthony Kelly, Abhishek Abhishek, Ana M. Valdes

**Affiliations:** 1 National Institute for Health Research, Nottingham Biomedical Research Centre, School of Medicine, University of Nottingham, Nottingham, United Kingdom; 2 Department of Health Sciences, Lund University, Lund University, Sweden

## Abstract

**Importance:**

Osteoarthritis is a prevalent, debilitating, and costly chronic disease for which recommended first-line treatment is underused.

**Objective:**

To compare the effect of an internet-based treatment for knee osteoarthritis vs routine self-management (ie, usual care).

**Design, Setting, and Participants:**

This randomized clinical trial was conducted from October 2018 to March 2020. Participants included individuals aged 45 years or older with a diagnosis of knee osteoarthritis recruited from an existing primary care database or from social media advertisements were invited. Data were analyzed April to July 2020.

**Interventions:**

The intervention and control group conformed to first-line knee osteoarthritis treatment. For the intervention group, treatment was delivered via a smartphone application. The control group received routine self-management care.

**Main Outcomes and Measures:**

The primary outcome was change from baseline to 6 weeks in self-reported pain during the last 7 days, reported on a numerical rating scale (NRS; range, 0-10, with 0 indicating no pain and 10, worst pain imaginable), compared between groups. Secondary outcomes included 2 physical functioning scores, hamstring and quadriceps muscle strength, the Western Ontario and McMaster Universities Osteoarthritis Index (WOMAC), and quantitative sensory testing.

**Results:**

Among a total of 551 participants screened for eligibility, 146 were randomized and 105 were analyzed (mean [SD] age, 66.7 [9.2] years, 71 [67.1%] women), including 48 participants in the intervention group and 57 participants in the control group. There were no significant differences in baseline characteristics between the groups. At the 6-week follow-up, the intervention group showed a greater NRS pain score reduction than the control group (between-group difference, −1.5 [95% CI, −2.2 to −0.8]; *P* < .001). Similarly, the intervention group had better improvements in the 30-second sit-to-stand test (between-group difference, 3.4 [95% CI, 2.2 to 4.5]; *P* < .001) and Timed Up-and-Go test (between-group difference, −1.8 [95% CI, −3.0 to −0.5] seconds; *P* = .007), as well as the WOMAC subscales for pain (between-group difference, −1.1 [95% CI, −2.0 to −0.2]; *P* = .02), stiffness (between-group difference, −1.0 [95% CI, −1.5 to −0.5]; *P* < .001), and physical function (between-group difference, −3.4 [95% CI, −6.2 to −0.7]; *P* = .02). The magnitude of within-group changes in pain (*d* = 0.83) and function outcomes (30 second sit-to-stand test *d* = 1.24; Timed Up-and-Go test *d* = 0.76) in the intervention group corresponded to medium to very strong effects. No adverse events were reported.

**Conclusions and Relevance:**

These findings suggest that this internet-delivered, evidence-based, first-line osteoarthritis treatment was superior to routine self-managed usual care and could be provided without harm to people with osteoarthritis. Effect sizes observed in the intervention group corresponded to clinically important improvements.

**Trial Registration:**

ClinicalTrials.gov Identifier: NCT03545048

## Key Points

**Question:**

What is the effectiveness of an internet-based exercise program vs routine self-management on pain outcomes among patients with knee osteoarthritis?

**Findings:**

This randomized clinical trial including 105 patients compared an internet-based program, including recommended information and exercises, with usual care for patients with knee osteoarthritis. Patients receiving the internet-based program experienced decreased pain and improved function at 6 weeks vs the usual care group.

**Meaning:**

These finding suggest that digitally delivered treatment information provided an important patient benefit and may decrease the burden of treatment for knee osteoarthritis on both patients and health care systems.

## Teaser

This randomized clinical trail examines the effectiveness of internet-based exercises aimed at treating knee osteoarthritis (iBEAT-OA) vs usual care for pain in patients with knee osteoarthritis.

## Introduction

Osteoarthritis is the most common joint disease and among the most prevalent chronic conditions.^1^ It causes pain and disability in adult and elderly populations, representing a heavy burden on health care systems and society. In the United Kingdom, as in rest of the Western world, 10% to 15% of adults consult their general practitioners about osteoarthritis every year.^1,2,3,4^ The UK National Institute for Health and Care Excellence (NICE) recommends that the first-line treatment for knee osteoarthritis should include disease information and a long-term exercise program.^5^

Long-term treatment in chronic diseases is not compatible with the need for cutting health care spending or for reducing face-to-face consultations during the ongoing coronavirus disease 2019 (COVID-19) pandemic. Therefore, internet-based remote treatment may be an efficacious and cost-effective alternative compared with routine in-person treatment.^6^ The advantages of internet-delivered health care in user flexibility and the ability to receive care at home (thus avoiding travel) have prompted the development of an internet-based first-line osteoarthritis management program consisting of exercises, informational lessons, an asynchronous dialogue with a physiotherapist, and outcome monitoring. A 2017 observational study^7^ reported improved physical function and decreased pain levels at 6 weeks among patients receiving an internet-based exercise program, and these improvements were confirmed at the 48-week follow-up.^8^ Additionally, some studies have shown that participants who took part in the 6-week digital program changed their mind regarding undergoing surgical treatment and expressed support for digital osteoarthritis treatment.^9,10,11^ However, no randomized clinical trials have compared this digital program with usual care. Previous studies using online interventions suggest that there is a lack of high-quality studies without methodological flaws (eTable 1 in Supplement 1); hence, there is a need for a randomized clinical trial assessing a digital intervention for knee osteoarthritis vs usual care. The aim of this study was to determine the efficacy of a 6-week internet-based exercise intervention^7^ to modulate pain, muscle strength, and function in individuals with knee osteoarthritis compared with self-managed usual care.

## Methods

This randomized clinical trial was approved by the sponsors and performed according to the Declaration of Helsinki.^12^ The study received approval from the Research Ethics Committee of Nottingham University, Health Research Authority, and the Nottingham University Hospitals National Health Service Trust Research and Innovation department. All participants provided written informed consent. This study is reported following the Consolidated Standards of Reporting Trials (CONSORT) reporting guideline.

### Trial Design

The Internet-Based Exercise Programme Aimed at Treating Knee Osteoarthritis (iBEAT-OA) is a parallel-group randomized clinical trial, performed in the primary care setting and managed from Nottingham City Hospital, Nottingham, United Kingdom, in participants with knee osteoarthritis, comparing a digitally delivered intervention with usual care self-management randomized in a 1-to-1 ratio. The trial protocol has been published elsewhere^13^ and can be found in Supplement 2.

### Participants

Participants were recruited from an existing database of community-dwelling adults with knee pain who had previously agreed to be contacted for future studies on osteoarthritis or from answering advertisements posted on social media.

Inclusion criteria were age 45 years or older, a clinical diagnosis of knee osteoarthritis (defined as knee pain for ≥3 months, early morning stiffness <30 minutes, crepitus, bony tenderness, and no palpable warmth),^14^ as well as radiographically established knee osteoarthritis (determined by Kellgren and Lawrence grade ≥1 on the 1-4 scale).^15,16^ Further inclusion criteria were the ability to read and write English and having access to and ability to use a smartphone or tablet.

Exclusion criteria were inability to give informed consent, terminal or mental illness, neurological conditions (ie, stroke, multiple sclerosis, Parkinson disease, motor neuron disease, muscular dystrophy, or Huntington disease), inflammatory joint diseases (eg, rheumatoid arthritis, gout, or calcium pyrophosphate deposition disease), or dementia. Further exclusion criteria were having been diagnosed with sleep apnea by physician, acute soft tissue injury to the knee within last 3 months before inclusion, and unstable heart condition or rapid fluctuations in hypertension, or a body mass index (BMI; calculated as weight in kilograms divided by height in meters squared) greater than 50.

### Study Groups

Both study arms relied on osteoarthritis self-management programs. The patients in the control arm received usual care, including exercise and information in accordance to the NICE guidelines^5^ and Versus Arthritis,^17^ delivered by their general practitioner and physiotherapists. The intervention group had a structured exercise and osteoarthritis disease information program delivered digitally via a smartphone application (hereafter, *app*) provided by Joint Academy.

#### Intervention Group

The treatment to the intervention group consisted of a 6-week digitally delivered program accessed via an iOS (Apple) or Google Play (Alphabet) app. It provided the intervention group with daily exercises and informative texts. The open- and closed-chain exercise instructions focused on neuromuscular leg strengthening and core stability and performance, as well as balance enhancement, as exemplified by doing sit-to-stand and stair-climbing exercises. These exercises were adjusted by the program in regard to degrees of complexity, load, and difficulty in relation to each participant’s response after doing the exercise, classified as too easy, good, or too difficult.^10^ The educational sessions covered the basics of osteoarthritis, its treatment, self-managing symptoms, the benefits of behavioral change and maintaining a healthy lifestyle. Each educational session was followed by a quiz to ensure that participants had understood the key messages. Adherence was encouraged by daily emails or smartphone notifications, or by the physiotherapist via asynchronous chat or telephone during the study period.

#### Usual Care Group

The usual care group was advised to continue with management of knee osteoarthritis as recommended by their general practitioner prior to trial recruitment. This involves use of core and adjunctive treatments, per NICE guidelines,^5^ and a self-management plan was developed, with patients able to initiate further consultations with general practitioners and therapists and referred to hospital specialists as required. Participants in the usual care group could continue to seek health care input for their knee pain as required during the duration of their study participation. Additionally, some participants in the usual care group were given a patient information leaflet on knee osteoarthritis developed by Versus Arthritis^17^ by their general practitioner or therapist.

### Outcomes

All participants were monitored similarly and had a face-to-face meeting at enrollment (ie, baseline) and after 6 weeks (ie, end of study) to assess function, muscle strength, and pain sensitization and to complete questionnaires. At the first session, participants who had not had a radiographic examination in the previous 12 months underwent standing radiographic imaging (posteroanterior view) for Kellgren and Lawrence grading.^15,16^ Participants were asked to provide information on analgesic medication use (over the counter or prescribed) at both sessions.

### Monitoring

The intervention group was actively monitored via the app for their exercise adherence, weight monitoring, and progress to next set of exercises. Additionally, support by a physiotherapist was available as needed (online or via telephone). In comparison, the usual care group was not monitored, motivated, or supported by the research physiotherapist.

Following randomization and allocation, participants in the intervention group were asked to answer the standard online questionnaire in the digital program covering baseline information, such as joint pain intensity, health-related quality of life, and physical function, as well as performing physical tests. The answer to this questionnaire established a baseline fitness level of participants to tailor the exercises (number of repetitions, specific performance). The results of online questionnaire to establish their baseline fitness level are not a part of assessment for this study.

### Primary End Point

The primary outcome was the between-group difference in change of participant-reported pain assessed by the question “How much pain have you had in the past week in your most painful knee? Can you kindly mark it on this scale?” reported on a numerical rating scale (NRS; range, 0-10, with 0 indicating no pain and 10, the worst pain imaginable)^18,19^ between baseline and 6 weeks.

### Secondary Outcomes

Secondary outcomes included the between-group differences in change of the Western Ontario and McMaster Universities Osteoarthritis Index (WOMAC), 2 physical functioning tests (ie, the 30-second sit-to-stand test and the Timed Up-and-Go [TUG] test), the Arthritis Research UK Musculoskeletal Health Questionnaire (MSK-HQ), maximum voluntary contraction of quadriceps and hamstring muscles, as well as quantitative sensory testing between baseline and 6 weeks.

The WOMAC self-administered questionnaire is a widely used, validated participant-reported outcome in hip and knee osteoarthritis.^20,21,22,23^ It contains 24 items divided into 3 subscales and scored on a scale of 0 to 4, with lower scores indicating lower levels of symptoms or physical disability, including pain (5 items; range, 0-20), stiffness (2 items; range, 0-8), and physical function (17 items; range, 0-68).

The 30-second sit-to-stand test was assessed as the number of times the participant could rise from a sitting position on a chair to a full standing position in 30 seconds, and the TUG test was assessed as the time (measured in seconds) required for the participant to stand up on therapist’s command, walk 3 m, turn around, walk back to the chair, and sit down again. Both are validated methods used to assess participants’ physical function.^24,25,26,27,28,29,30,31,32^ At enrollment, participants had a demonstration of the tests and practiced once before doing each test. The TUG was repeated 3 times and the mean time was used. The 30-second sit-to-stand test was conducted once to avoid fatigue. A break was allowed between both tests.

The MSK-HQ is a self-administered validated questionnaire allowing people with musculoskeletal conditions to report symptoms and quality of life in a standardized way, including stiffness, generalized well-being, difficulty with sleeping, and understanding of the diagnosis and treatment.^33^ The 14 questions are scored on a scale of 0 to 4 (range, 0-54), with lower scores indicating lower levels of symptoms.

Isokinetic peak torque of quadriceps and hamstring muscles was measured as newton meters (Nm) at 60°/s and 180°/s using a HUMAC / NORM Testing and Rehabilitation System model 7709 (Computer Sports Medicine ). A standardized protocol^34^ was used and is a valid and reliable assessment method of muscle strength in quadriceps and hamstrings.^35^

The quantitative sensory testing included pressure pain threshold (PPT) to measure tenderness around the knee joint (with 0 indicating maximum tenderness),^36,37,38,39^ temporal summation (TS) to measure element of central sensitization (range, 0-10, with 10 indicating maximum sensitization),^40,41,42,43^ and conditional pain modulation (CPM) to assess the function of endogenous pain inhibitory pathways in humans (with 0 indicating maximum inhibitory pathways).^44,45,46,47^ In addition, sleeping assessment with actigraphy, Pittsburgh Sleep Quality Index, musculoskeletal ultrasonographic assessment, and serum biomarkers of inflammation were measured and will be reported separately in future studies.

#### Problems and Harms

Participants were informed via the participant information sheet required by Health Research Authority,^48^ that if they have any concerns, they should speak to one of us (S.G.). Any adverse event or harm would be escalated to principal investigator (A.V.) and ultimately reported to the sponsor of this study.

#### Sample Size

A systematic review of 44 high-quality exercise trials for knee osteoarthritis pain (3537 participants) found a mean effect of 12 of 100 points on a visual analogue scale corresponding to a standardized effect size (Cohen *d*) of 0.49.^49^ Based on this estimated effect size (corresponding to 1.2 points on the NRS scale), a sample size of 60 participants per group was needed to achieve 75% power, with α level set to .05. The estimated dropout rate for exercise interventions was 12%; therefore, a sample of 67 participants per group was planned.

#### Randomization

Participants were randomized by the research team using Sealed Envelope randomization software version 1.19.1 at a 1-to-1 ration for the intervention or usual care. The code break was kept secure by the research manager.

#### Blinding and Similarities of Interventions

Owing to the nature of the study, no blinding of participants or observers occurred. The intervention and usual care protocols followed the NICE management recommendations for knee osteoarthritis.^50^

### Statistical Analysis

Analyses were performed as intention-to-treat, defined as all participants randomly assigned to a treatment group and being assessed after randomization. Any missing values of questionnaire items for participants who completed both baseline and 6 week follow up sessions were imputed using mean imputation method for both groups (eTable 2 in Supplement 1).

Baseline and follow-up scores are presented for each group as means and SDs, since there were no extreme outliers affecting those measures. For each outcome variables, a change score between baseline and the 6-week follow-up was calculated and used as the outcome variables in the comparison between groups. Since the change scores followed an approximately normal distribution, comparisons between the intervention and control group were performed by analysis of variance, adjusted for baseline scores (analysis of covariance).^51^ Sex, age, BMI, Kellgren and Lawrence grade, and use of medication were included in the models to evaluate potential confounding that could have occurred from unbalanced distribution between groups, despite randomization. The evaluation was based on a change in estimate approach.^52^ Since the inclusion did not result in any change in estimate more than 15%, the variables were excluded from the final analyses. Estimated mean differences in change scores between the groups, adjusted for baseline scores, are presented with accompanying 95% CIs. Within-group changes were evaluated by estimated mean change with accompanying 95% CIs. For within-group comparison, statistical significance was determined by 95% CIs not crossing 0. Standardized effect sizes (Cohen *d*) were computed for within-group changes as observed mean change baseline-follow up divided by pooled SD ([*baseline SD* + *follow-up SD*] / 2). The data were analyzed using SPSS statistical software version 24 (IBM) by an author who was unaware of group assignment (F.E.), per the statistical analysis plan in Supplement 2.

## Results

**Figure 1.**
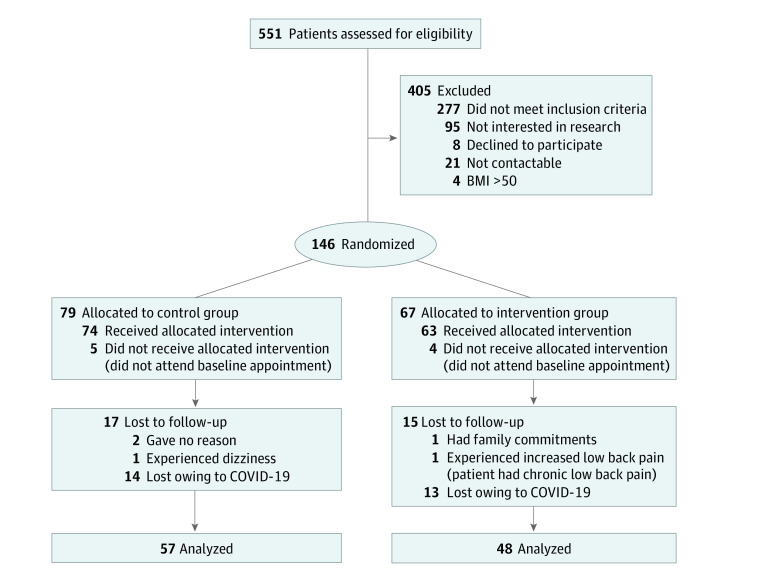
Flowchart of Participants BMI indicates body mass index (calculated as weight in kilograms divided by height in meters squared); COVID-19, coronavirus disease 2019.

The trial started in late 2018. The control and intervention participant flowchart, including exclusions and losses to follow-up, are shown in Figure 1. Among a total of 551 participants screened for eligibility, 146 were randomized, including 79 participants randomized to the intervention group and 67 participants randomized to the usual care group. Nine participants in each group did not attend the baseline appointment, and no baseline data and demographic characteristics were obtained for these participants. Owing to the COVID-19 lockdown in the United Kingdom in March 2020, the study ended before 27 participants had their face-to-face follow-up visit at 6 weeks (Figure 1). In addition, 3 participants in the usual care group and 2 participants in the intervention group were lost to follow-up (Figure 1) and were therefore excluded from the analyses. A total of 105 participants were analyzed (mean [SD] age, 66.7 [9.2] years, 71 [67.1%] women), including 48 participants in the intervention group (mean [SD] age, 65.2 [9.7] years; 34 [70.8%] women; mean [SD] BMI, 30.4 [5.5]) and 57 participants in the usual care group (mean [SD] age, 68.0 [8.6] years; 37 [64.9%] women; mean [SD] BMI, 31.9 [5.9]) completed this study. No significant difference was observed between participants groups in terms of use of analgesic medications before or after the intervention. In the intervention group, the mean (SD) adherence with the internet-based exercise program was 87.9% (14.3%) of sessions completed.

### 

#### Baseline Data

The mean baseline demographic and clinical variables were similar for participants in both groups (Table 1). The mean baseline demographic and clinical variables for 17 participants in the control group and 15 participants in the intervention group who could not finish follow-up session were similar to the participants who completed both sessions (Table 1). Owing to COVID-19 lockdown restrictions, baseline data for WOMAC and MSK-HQ for these 32 participants were not retrieved for analysis.

**Table 1.** Participant Baseline Characteristics

Characteristic	Participants, mean (SD)			
	Usual care (n = 57)	Intervention (n = 48)	Withdrew^a^	
			Usual care (n = 17)	Intervention (n = 15)
Age, y	68.0 (8.6)	65.2 (9.7)	64 (8)	64 (5)
Sex, No (%)				
Women	37 (64.9)	34 (70.8)	11 (64.7)	11 (73.3)
Men	20 (35.1)	14 (29.2)	6 (35.3)	4 (26.7)
BMI	31.9 (5.9)	30.4 (5.5)	28.7 (4.6)	30.5 (4.9)
Radiographic Score, No. (%)^14,15^	2.1	2.0	1.9	1.9
1	18 (31.6)	19 (39.6)	6 (35.3)	5 (33.3)
2	22 (38.6)	13 (27.1)	8 (47.1)	7 (46.7)
3	11 (19.3)	11 (22.9)	2 (11.8)	2 (13.3)
4	6 (10.5)	5 (10.4)	1 (5.9)	1 (6.7)
NRS pain^b^	4.7 (2.1)	4.4 (2.0)	4.3 (1.5)	4.3 (1.6)
WOMAC^c^				
Pain	7.8 (3.7)	8.0 (3.9)	NA	NA
Stiffness	3.1 (1.6)	4.0 (1.7)	NA	NA
Physical function	28.3 (12.8)	26.8 (12.9)	NA	NA
TUG, s^d^	9.9 (3.6)	9.0 (1.4)	9.1 (2.5)	9.4 (1.9)
30-s sit-to-stands, No.^e^	9.2 (4.3)	9.3 (2.7)	10.1 (1.7)	9.8 (1.9)
MSK-HQ^f^	28.4 (10.1)	30.7 (9.9)	NA	NA
PPT				
Superolateral patella	317.3 (170.2)	314.4 (153.7)	350.1 (84.8)	315.6 (150.7)
Superomedial patella	334.1 (147.4)	326.0 (149.9)	370.5 (131.7)	305.4 (103.8)
Medial joint line	355.1 (202.5)	318.7 (159.0)	359.7 (173.0)	319.0 (105.4)
Tibialis anterior muscle	367.0 (182.4)	386.40 (175.8)	372.8 (150.2)	373.0 (122.8)
Temporal summation	1.8 (1.8)	2.30 (1.8)	1.9 (1.1)	2.2 (1.7)
Conditional pain modulation	52.8 (110.7)	53.4 (97.6)	41.0 (130.4)	77.5 (100.5)
Isokinetic peak torque, Nm^g^				
Quadriceps 60°/s	69.4 (42.7)	79.52 (45.6)	77.7 (29.8)	80.1 (37.7)
Hamstring 60°/s	52.19 (26.7)	54.44 (29.6)	60.5 (23.0)	63.3 (25.3)
Quadriceps 180°/s	40.7 (26.1)	46.10 (28.8)	53.1 (24.0)	49.7 (22.1)
Hamstring 180°/s	37.3 (21.2)	40.8 (22.5)	47.9 (13.3)	45.2 (14.2)

Abbreviations: BMI, body mass index (calculated as weight in kilograms divided by height in meters squared); MSK-HQ, Musculoskeletal Health Questionnaire; NA, not applicable; NRS, numerical rating scale; PPT, pressure pain threshold; TUG, Timed Up-and-Go test; WOMAC, Western Ontario and McMaster Universities Osteoarthritis Index.

^a^ Includes participants who dropped out or who were unable to have a second assessment after 6-weeks because of the UK lockdown on March 23, 2020.

^b^ Range, 0 to 10, with 0 indicating no pain and 10, the worst pain imaginable.

^c^ Range, 0-4, with lower scores indicating lower levels of pain (5 items; range, 0-20), stiffness (2 items; range, 0-8), and physical function (17 items; range, 0-68).

^d^ Measured in seconds, the participant stands up at therapist’s command, walks 3 m, turns around, walks back to the chair and sits down.

^e^ Counts the number of times the participant comes from a sitting position on a chair to a full standing position in 30 seconds.

^f^ Scored on a scale of 0 to 4, with lower scores indicating lower levels of symptoms or physical disability; the total score is the sum of all items.

^g^ The isokinetic peak torque (Newton meter) of quadriceps and hamstring muscles measured at 60°/s and at 180°/s.

#### Primary Outcome

**Figure 2.**
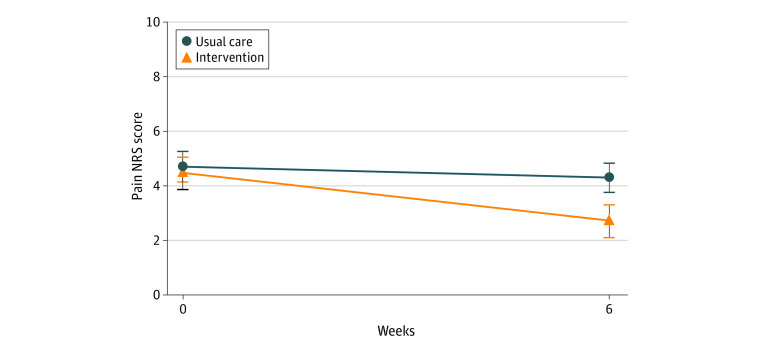
Change in Numerical Rating Scale (NRS) Pain Scores From Baseline to 6 Weeks

The intervention group showed a greater decrease in NRS pain score from baseline to 6 weeks compared with the control group (between-group difference, −1.5 [95% CI, −2.2 to −0.8]; *P* < .001) (Table 2; Figure 2). Between baseline and the 6-week follow-up, there was a statistically significant improvement in NRS pain scores in the intervention group (mean change, −1.8 [95% CI, −2.4 to −1.3]; *d* = −0.83) but not in the usual care group (mean change, −0.3 [95% CI, −0.8 to 0.2]; *d* = −0.2) (Figure 2).

**Table 2.** Change in Primary and Secondary Outcomes

Outcome	Usual care group		Intervention group		Between-group difference in change, mean (95% CI)^a^	*P* value
	Change, mean (95% CI)	Cohen *d*, change	Change, mean (95% CI)	Cohen *d*, change		
NRS pain^b^	−0.3 (−0.8 to 0.2)	−0.20	−1.8 (−2.4 to −1.3)	−0.83	−1.5 (−2.2 to 0.8)	<.001
WOMAC^c^						
Pain	−1.2 (−1.8 to −0.5)	−0.33	−2.2 (−2.9 to −1.6)	−0.60	−1.1 (−2.0 to −0.2)	.02
Stiffness	0.2 (−0.1 to 0.6)	0.24	−0.8 (−1.2 to −0.4)	−0.51	−1.0 (−1.5 to −0.5)	<.001
Physical function	−4.3 (−6.2 to −2.4)	−0.37	−7.8 (−9.8 to −5.7)	−0.60	−3.4 (−6.2 to −0.7)	.02
TUG, s^d^	0.4 (−0.5 to 1.2)	0.03	−1.4 (−2.3 to −0.5)	−0.76	−1.8 (−3.0 to −0.5)	.007
30-s sit-to-stand test, No.^e^	1.2 (0.4 to 2.0)	0.26	4.5 (3.7 to 5.4)	1.24	3.4 (2.2 to −4.5)	<.001
MSK-HQ^f^	1.6 (−0.4 to 3.6)	0.14	1.2 (−0.9 to 3.4)	0.11	−0.3 (−3.3 to 2.6)	.82
PPT						
Superolateral patella	−55.6 (−86.1 to −25.2)	−0.36	−30.5 (−63.7 to 2.6)	−0.20	25.1 (−19.9 to 70.1)	.27
Superomedial patella	−44.0 (−72.4 to −15.7)	−0.32	−22.3 (−53.2 to 8.7)	−0.14	21.8 (−20.2 to 63.9)	.31
Medial joint line	−49.1 (−81.4 to −16.7)	−0.31	−18.9 (−54.1 to 16.4)	−0.06	30.2 (−17.8 to 78.2)	.22
Tibialis anterior muscle	−54.6 (−86.1 to −23.0)	−0.30	−11.4 (−45.8 to 23.0)	−0.09	43.2 (−3.6 to 89.9)	.07
Temporal summation	0.1 (−0.3 to 0.5)	0.11	−0.1 (−0.5 to 0.4)	−0.18	−0.1 (−0.7 to 0.4)	.63
Conditional pain modulation	−4.9 (−29.2 to 19.5)	−0.04	−23.8 (−50.3 to 2.8)	−0.26	−18.9 (−55.0 to 17.2)	.30
Isokinetic peak torque, Nm^g^						
Quadriceps 60°/s	3.9 (−0.8 to 8.7)	0.10	9.6 (4.4 to 14.7)	0.20	5.7 (−1.4 to −12.7)	.11
Hamstring 60°/s	3.6 (−0.02 to 7.2)	0.13	10.5 (6.6 to 14.6)	0.35	6.9 (1.6 to 12.3)	.01
Quadriceps 180°/s	2.5 (−1.5 to 6.5)	0.10	5.1 (0.8 to 9.5)	0.17	2.7 (−3.3 to 8.6)	.38
Hamstring 180°/s	4.0 (1.0 to 7.1)	0.18	5.8 (2.5 to 9.1)	0.26	1.8 (−2.7 to 6.3)	.43

Abbreviations: MSK-HQ, Musculoskeletal Health Questionnaire; NRS, numerical rating scale; PPT, pressure pain threshold; TUG, Timed Up-and-Go test; WOMAC, Western Ontario and McMaster Universities Osteoarthritis Index.

^a^ Adjusted for baseline score.

^b^ Range, 0 to 10, with 0 indicating no pain and 10, the worst pain imaginable.

^c^ Range, 0-4, with lower scores indicating lower levels of pain (5 items; range, 0-20), stiffness (2 items; range, 0-8), and physical function (17 items; range, 0-68).

^d^ Measured in seconds, the participant stands up at therapist’s command, walks 3 m, turns around, walks back to the chair, and sits down.

^e^ Counts the number of times the participant comes from a sitting position on a chair to a full standing position in 30 seconds.

^f^ Scored on a scale of 0 to 4, with lower scores indicating lower levels of symptoms or physical disability; the total score is the sum of all items.

^g^ The isokinetic peak torque (Newton meter) of quadriceps and hamstring muscles measured at 60°/s and at 180°/s.

#### Secondary Outcomes

The between-group analysis of mean change from baseline to 6 weeks showed that the intervention group improved statistically significantly more than the control group in the WOMAC subscales for pain (between-group difference, −1.1 [95% CI, −2.0 to −0.2]; *P* = .02), stiffness (between-group difference, −1.0 [95% CI, −1.5 to −0.5]; *P* < .001) and physical function (between-group difference, −3.4 [95% CI, −6.2 to −0.7]; *P* = .02), the 30-second sit-to-stand test (between-group difference, 3.4 [95% CI, 2.2 to 4.5]; *P* < .001), the TUG test (between-group difference, −1.8 [95% CI, −3.0 to −0.5] seconds; *P* = .007), and hamstring isokinetic strength at 60°/s (between-group difference, 6.9 [95% CI, 1.6 to 12.3] Nm; *P* = .01). There were no statistically significant between-group differences regarding change in remaining strength measures, PPT, TS, CPM, or MSK-HQ.

**Figure 3.**
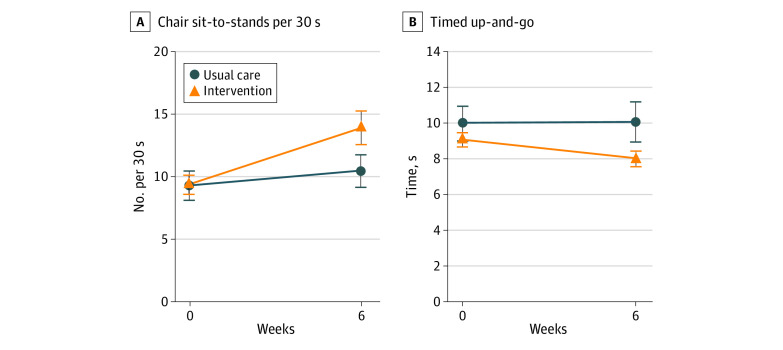
Change in 30-Second Chair Sit-to-Stand and Timed Up-and-Go Scores From Baseline to 6 Weeks

The within-group mean changes showed that the intervention group improved significantly in the 3 WOMAC subscales (pain change, −2.2 [95% CI, −2.9 to −1.6]; *d* = −0.60; stiffness change, −0.8 [95% CI, −1.2 to −0.4]; *d* = −.51; and function change, −7.8 [95% CI, −9.8 to −5.7]; *d* = −0.60), 30-second sit-to-stand test (change, 4.5 [95% CI, 3.7 to 5.4]; *d* = 1.24), TUG test (change, −1.4 [95% CI, −2.3 to −0.5] seconds; *d* = −0.76), quadriceps isokinetic strength at 60°/s (change, 9.6 [95% CI, 4.4 to 14.7] Nm; *d* = 0.20), and hamstring isokinetic strength at 60°/s (change, 10.5 [95% CI, 6.6 to 14.6] Nm; *d* = 0.35) and at 180°/s (change, 5.8 [95% CI, 2.5 to 9.1] Nm; *d* = 0.26) (Table 2, Figure 3). For the usual care group between baseline and follow up, there was a statistically significant change in WOMAC subscale scores for pain (change, −1.2 [95% CI, −1.8 to −0.5]; *d* = −0.33) and function (change, −4.3 [95% CI, −6.2 to −2.4]; *d* = −0.37), PPT (superolateral patella change, −55.6 [95% CI, −86.1 to −25.2]; *d* = −0.36; superomedial patella change, −44.0 [95% CI, −72.4 to −15.7]; *d* = −0.32; medial joint line change, −49.1 [95% CI, −81.4 to −16.7]; *d* = −0.31]; tibialis anterior muscle, −54.6 [95% CI, −86.1 to −23.0]; *d* = −0.30), and the 30-second sit-to-stand test (change, 1.2 [95% CI, 0.4 to 2.0]; *d* = 0.26) (Table 2). There were no within-group changes in TS, CPM, or MSK-HQ questionnaire scores in any of the groups. No serious adverse events were reported in any of the study groups.

## Discussion

In this randomized clinical trial, we found that an internet-based first-line knee osteoarthritis management program was superior to routine self-managed care in the primary outcome, NRS pain score, as well as secondary outcomes of function performance. Participants in both groups were treated in accordance with guidelines.^5,53^

Between-group differences in change from baseline to 6 weeks suggest a clinically relevant benefit resulting from participation in the internet-based program compared with the self-management program. The minimally important change between treatments and responder criteria are debated concepts challenging their application.^54,55,56^ However, the improvements seen in the internet-based intervention group, with standardized effect sizes (ie, Cohen *d*) corresponding to medium to very strong effects, strongly suggest a clinically important improvement. In comparison, the improvements among participants in the self-managed usual care group were small or absent, with standardized effect sizes corresponding to minor or weak effects. The effect sizes attained with the internet-based program used in this study are comparable with or greater than those presented in systematic reviews of face-to-face exercise programs.^49,57^

The internet-based program used in this study was derived from the better management of patients with osteoarthritis (BOA) program, initiated in Sweden in 2008 to implement existing osteoarthritis treatment guidelines for education and exercise face-to-face in primary care facilities.^58,59^ Observational studies from the BOA and Good Life with Osteoarthritis in Denmark (GLA:D) programs subsequently launched in Denmark and other countries reported that among patients with knee and hip osteoarthritis, participating in these programs was associated with reductions in symptoms, improved function, decrease in willingness to undergo surgical treatment, decrease in use of osteoarthritis medication, and reduced use of sick leave.^58,59,60^

The factors associated with the superiority of the internet-based program vs self-management in this study are not clear. Possibly, daily delivery of individualized treatment, together with support, engagement, and nudging from health care professionals, may have played a role.^7,8,10^ Other studies have suggested that increasing exercise frequency, but not necessarily length of sessions, can improve pain relief and that exercise should be performed at least 3 times a week.^57,61^ The superior outcome in the intervention group may depend on the content and context in the app, including a combination of standardized exercises and information, as well as using a digital delivery system.

Our results are consistent with studies showing efficacy and effectiveness of digital management for other chronic conditions.^62,63,64^ Advantages of a digitally delivered treatment may include lower costs and easier access for patients living in remote areas where transport may be an added obstacle. In a pandemic, such as the ongoing COVID-19 pandemic, digitally delivered care can continue without interruption.

### Generalizability

The demographic and basic osteoarthritis characteristics of participants in our randomized clinical trial (ie, age, sex, pain, function) were similar to participants in the population-based osteoarthritis registers of BOA, GLA:D, and Joint Academy.^7,8,59,60^ Likewise, patients eligible for total knee joint replacement randomized to operation or first-line treatment, had similar average pain level as in the present study.^66^

### Limitations

This study has some limitations. Owing to the COVID-19 lockdown, 27 participants could not attend the follow-up visit, preventing the study from reaching the planned statistical power. A further 5 participants discontinued treatment, but this loss to follow-up was less than the expected loss of 12%. Numbers of participants not attending the 6-week follow-up were similar in both groups, and they had similar demographic and baseline characteristics compared with the analyzed set of participants. Additionally, blinding was not possible for this study, and potential placebo effects associated with internet-based treatment compared with usual care are not accounted for.

## Conclusions

This parallel-group randomized clinical trial compared knee osteoarthritis management via a digital platform with a self-management usual care program. Both forms of management conformed to clinical guidelines. Digital delivery was superior to routine self-management. No serious harms were reported. Our findings suggest that digital treatment has the potential to decrease the osteoarthritis burden on both the health care systems and patients.^65^

## Notes

Supplement 1. **eTable 1.** Studies on Internet and Web-Based Exercises for Managing Knee Osteoarthritis **eTable 2.** Missing Data Itemized

Supplement 2. **Trial Protocol and Statistical Analysis Plan**

Supplement 3. **Data Sharing Statement**
