# Error in Visual Abstract

**DOI:** 10.1001/jamanetworkopen.2021.6209

**Published:** 2021-03-25

In the Original Investigation titled “Effectiveness of Internet-Based Exercises Aimed at Treating Knee Osteoarthritis: the iBEAT-OA Randomized Clinical Trial,”^1^ published February 23, 2021, the results in the visual abstract for the intervention and control groups were transposed. These should have appeared as usual care: −0.3 (95% CI, −0.8 to 0.2); intervention: −1.8 (95% CI, −2.4 to −1.3). This article has been corrected.^1^
