# Supplementary material for: Effectiveness of Internet-Based Exercises Aimed at Treating Knee Osteoarthritis: The iBEAT-OA Randomized Clinical Trial
Source: JAMA Netw Open. 2021 Feb 23;4(2):e210012. doi: 10.1001/jamanetworkopen.2021.0012 (PMC7903254; doi:10.1001/jamanetworkopen.2021.0012)
Supplement: Supplement 1. — eTable 1. Studies on Internet and Web-Based Exercises for Managing Knee Osteoarthritis eTable 2. Missing Data Itemized [file jamanetwopen-e210012-s001.pdf]

Supplemental Online Content

Gohir SA, Eek F, Kelly A, Abhishek A, Valdes AM. Effectiveness of internet-based exercises aimed at treating knee osteoarthritis: the iBEAT-OA randomized clinical trial. *JAMA Netw Open*. 2021;4(2):e210012. doi:10.1001/jamanetworkopen.2021.0012

eTable 1. Studies on Internet and Web-Based Exercises for Managing Knee Osteoarthritis

eTable 2. Missing Data Itemized

This supplemental material has been provided by the authors to give readers additional information about their work.

© 2021 Gohir SA et al. *JAMA Network Open.*

eTable 1: Studies on Internet and web-based exercises for managing knee osteoarthritis.

Author, publication year And location	Study Type	Fully online	Online + Face to Face Session	X-rays	Primary outcome	Follow up duration	Limitations
**Lorig et al. (2008) USA**	Randomised Controlled trial (RCT)	YES	N/A	Not done	Six outcome measures; Pain, fatigue, activity limitation, health distress, disability, and self-reported global health	6 months and 12 months	• Participants had osteoarthritis, rheumatoid arthritis or fibromyalgia (not specific to knee OA)<br>• differences in change scores all favoured the treatment group
**Bossen et al. (2013) Netherland**	Randomised Controlled trial (RCT)	YES	N/A	Not done	Physical Activity	3 months and 9 months (9-week intervention)	• No X-rays<br>• Lack of exercise and participant' favourite recreational activity done<br>• Participants had knee and hip OA<br>• 15.6% dropout after 3 months and 24.6% after 12 months
**Brooks et al. (2014) USA**	Pilot study	YES	N/A	YES	WOMAC	8 weeks	• No control group<br>• Lacking participants with severe OA
**Dahlberg et al. (2016) Sweden**	Pilot study	YES	N/A	Not done	Pain (NRS)	6 weeks	• Not RCT<br>• No X-rays<br>• Online recruitment – population used to online medium?<br>• Small sample size
**Nero et al. (2017)**	Observational	YES	N/A	Not done	Pain (NRS)	6 weeks	• Not RCT<br>• No X-rays

© 2021 Gohir SA et al. *JAMA Network Open.*

**Sweden**	Quasi-Experimental Study						• Online recruitment – population used to online medium?
**Bennell et al. (2017) Australia**	Randomised Controlled trial (RCT)	YES	N/A	Not done	Pain (NRS)	3 months	• No X-rays<br>• Participants were not blinded to treatment<br>• No clinical examination<br>• Could no establish minimum number of online skype session for clinical effectiveness<br>• Participants in intervention group had higher educational level
**Kloek et al. (2018) Australia**	Randomised Controlled trial (RCT)	N/A	X<br>5 face to face session	Not done	Knee Injury and OA Outcome Score<br>And Timed up and go score	3 & 12 months	• No X-rays<br>• No clinical examination<br>• Hip & knee are included<br>• 15% dropout after 3 months and 35% after 12 months

© 2021 Gohir SA et al. *JAMA Network Open.*

eTable 2: Missing Data Itemized.

Variable	Sub-Variable	Data missing in control group (Baseline)	Data missing in control group (Follow up)	Data Missing in intervention group (Baseline)	Data Missing in intervention group (Follow up)
**NRS Pain**		0	0	0	0
**WOMAC**	Pain	0	0	0	0
	Stiffness	1	0	0	1
	Physical Function	2	1	0	0
**TUG**		0	0	0	0
**30CST**		0	0	0	0
**MSK-HQ**		1	1	2	1
**PPT**	Superolateral Patella	0	0	0	0
	Superomedial Patella	0	0	0	0
	Medial Joint line	0	0	0	0
	Tibialis Anterior Muscle	0	0	0	0
**Temporal Summation (TS)**		0	0	0	0
**Conditional Pain Modulation (CPM)**		0	0	0	0
**Isokinetic Peak Torque**	Q60	0	0	0	0
	H60	0	0	0	0
	Q180	0	0	0	0
	H180	0	0	0	0

NRS Pain, 0-10 where 0 is no pain and 10 is the worst pain imaginable. **WOMAC** items are scored on a scale of 0-4 (lower scores indicate lower levels of Pain (5 items, 0-20), stiffness (2 items, 0-8), and physical function (17 items, 0-68). **TUG** is measured in seconds, the participant stands up upon therapist's command, walks 3 meters, turns around, walks back to the chair and sits down. **30CST** Counts the number of times the participant comes from a sitting position on a chair to a full standing position in 30 seconds. **PPT, TS, CPM** (0 to maximum value). **The isokinetic peak torque** (Newton meter) of quadriceps and hamstring muscles measured at 60 degrees/second and at 180 degrees/second. **MSK-HQ**, 14 questions scored on a scale of 0-4 (lower scores indicate lower levels of symptoms or physical disability), the total score is the sum of all items.

© 2021 Gohir SA et al. *JAMA Network Open.*
