# Supplementary material for: Effectiveness of Internet-Based Exercises Aimed at Treating Knee Osteoarthritis: The iBEAT-OA Randomized Clinical Trial
Source: JAMA Netw Open. 2021 Feb 23;4(2):e210012. doi: 10.1001/jamanetworkopen.2021.0012 (PMC7903254; doi:10.1001/jamanetworkopen.2021.0012)
Supplement: Supplement 2. — Trial Protocol and Statistical Analysis Plan [file jamanetwopen-e210012-s002.pdf]

Gohir SA, Eek F, Kelly A, Abhishek A, Valdes AM. Effectiveness of internet-based exercises aimed at treating knee osteoarthritis: a randomized clinical trial. *JAMA Netw Open*. 2021;4(2):e210012. doi:10.1001/jamanetworkopen.2021.0012

A. Trial Protocol
B. Statistical Analysis Plan

This supplemental material has been provided by the authors to give readers additional information about their work.

A. Trial Protocol

Randomised controlled trial evaluating the efficacy of web-based exercises on knee pain due to knee osteoarthritis

Final Draft V 1.0
26th April 18

Short title: Effects of web-based exercises on the population with knee arthritis

Funding Source: Arthritis Research UK (ARUK) Pain Centre
and
MRC Arthritis Research UK Centre for MSK Ageing Research, School of Life Sciences, University of Nottingham

SYNOPSIS

Title	Effects of web-based exercises on the population with knee arthritis
Short title	Knee arthritis and exercises
Chief Investigator	Dr Ana M Valdes
Objectives	[1] To test whether internet-based exercises reduce the pain in knee OA<br>[2] To check whether internet-based exercises improve the physical activity in the patients with knee OA.<br>[3] To explore the correlation between sleep, knee inflammation (effusion, synovial hypertrophy or/and synovial hyper vascularity) and biomarkers of insulin resistance and knee pain.
Study Configuration	Randomised controlled trial
Setting	Primary care
Sample size estimate	Control and interventional arms each consisting of 67 individuals with predicted dropout rate of 10-12%<br>A sample size of n=60 per group is necessary to achieve 75% stat power. The estimated dropout rate for exercise interventions is 12%. Therefore, a sample of 67 per arm will be recruited to produce at least 75% power.
Number of participants	67 in each group and 134 in total
Eligibility criteria	• Arthritis in at least one knee with at least score of grade 1 or above osteoarthritis on Kellgren and Lawrence classification<br>• Age group > 45 years<br>• Able to use internet<br>• Able to read and write the English language
Description of interventions	20-30 minutes of gentle exercises on a daily basis for six weeks (interventional group)<br><br>Control group will continue with their routine practice – advice/support given by GP.
Duration of study	24 months<br>Planned recruitment date: April / May 2018
Randomisation and blinding	Computer-based randomisation<br>Pragmatic randomised controlled trial (RCT)
Outcome measures	**Primary:** Knee pain measured on Numerical Rating Scale (NRS).<br>**Secondary:**<br>Sleep

	Pain (Pressure pain threshold – PPT, Temporal summation – TS, and conditional pain modulation – CPM)<br>Muscle Strength - Dynamometer<br>Physical functioning (Time up and go test, 30-second sit to stand test)<br>Western Ontario and McMaster Universities Osteoarthritis Index (WOMAC)<br>General health questionnaire (MSK- HQ)
Statistical methods	Standard parametric regression methods will be used to assess the effect of exercise on pain sensitivity, pain scores, and sleep patterns

ABBREVIATIONS

ARUK	Arthritis Research United Kingdom
CI	Chief Investigator overall
RCT	Randomised controlled trial
CPM	Conditioned pain modulation
DAP	Data Analysis Plan
DNIC	Diffuse Noxious Inhibitory Control
GCP	Good Clinical Practice
HADS	Hospital Anxiety and Depression Scale
ICF	Informed Consent Form
JA	Joint Academy
K/L	Kellgren and Lawrence system for classification of osteoarthritis of knee
MSK-HQ	The arthritis Research UK Musculoskeletal Health Questionnaire
MSK-USS	Musculoskeletal ultrasound scan
MVC	Maximum Voluntary Contraction
NHS	National Health Service
NRS	Numerical rating scale
NUH	Nottingham University Hospital Trust
OA	Osteoarthritis
PA	Posterior-Anterior
PIS	Participant Information Sheet
PPI	Patient and Public Involvement
PSQI	Pittsburgh sleep quality index
QST	Quantitative Sensory Testing
REC	Research Ethics Committee
R&D	Research and Development department
SAE	Serious Adverse Event
SF	Synovial fluid

TS	Temporal Summation
TUG	Time up and go test
WOMAC	Western Ontario and McMaster Universities Osteoarthritis Index
30CST	30-second sit to stand test

TABLE OF CONTENTS

SYNOPSIS **3**

ABBREVIATIONS **5**

STUDY BACKGROUND INFORMATION AND RATIONALE **9**

STUDY OBJECTIVES AND PURPOSE **11**

PURPOSE 11
PRIMARY OBJECTIVE 11
SECONDARY OBJECTIVES 11

DETAILS OF PRODUCTS **11**

Description 11
Manufacture 11
Known Side Effects 12

STUDY DESIGN **12**

STUDY CONFIGURATION 12
RANDOMIZATION AND BLINDING 12
Maintenance of Randomisation Codes and Procedures for Breaking Code 12
STUDY MANAGEMENT 12
DURATION OF THE STUDY AND PARTICIPANT INVOLVEMENT 13
End of the Trial 13
SELECTION AND WITHDRAWAL OF PARTICIPANTS 13
Recruitment 13
Inclusion criteria: 14
Exclusion criteria: 14
Informed consent 14
STUDY REGIMEN 15
Muscle and Strength Measurements 16
Compliance 18
Criteria for terminating the study 18
TRANSPORT AND STORAGE OF THE TISSUES 19
LABORATORY ANALYSIS 19

STATISTICS **19**

Methods 19
Sample size and justification 19

ADVERSE EVENTS **20**

ETHICAL AND REGULATORY ASPECTS **20**

ETHICS COMMITTEE AND REGULATORY APPROVALS 20
INFORMED CONSENT AND PARTICIPANT INFORMATION 21
RECORDS 21
Study Forms 21
Sample Labelling 21
Source documents 22
Direct access to source data/documents 22
DATA PROTECTION 22

QUALITY ASSURANCE & AUDIT **22**

INSURANCE AND INDEMNITY 22
STUDY CONDUCT 22
STUDY DATA 22
RECORD RETENTION AND ARCHIVING 23
DISCONTINUATION OF THE TRIAL BY THE SPONSOR 23
STATEMENT OF CONFIDENTIALITY 23

PUBLICATION AND DISSEMINATION POLICY 23

USER AND PUBLIC INVOLVEMENT 23

STUDY FINANCES 24

SIGNATURE PAGES 25

REFERENCES 26

STUDY BACKGROUND INFORMATION AND RATIONALE

Osteoarthritis is the most common cause of disability in the elderly population and most individuals suffering from osteoarthritis is managed in the primary care setting (1). Knee osteoarthritis is a most common form of arthritis in the world (2). The rate of knee arthritis is as high as that of cardiac disease and is the most common problem in the individuals over the age of 65 (3). In the United Kingdom, 10% of 65-74-year-old individuals consult their general practitioners about osteoarthritis per year (4). 4% population attend their general practitioners as a result of knee osteoarthritis and half of them (2%) consult their general practitioner for the first time or with the acute flare of knee arthritis (1).

According to national and international guidelines, the first line treatment for osteoarthritis pain (OA) is non-surgical; exercise, information and, in relevant cases, weight loss (5-9). There is enough evidence for the effectiveness of exercises in the management of knee osteoarthritis and to improve the functional capacity of these individuals to cope better with the activities of daily living (10-24). In fact, one study reported a slowdown of progressive radiographic changes of knee OA as a result of strengthening exercises (25).There is a significant disparity on the effectiveness of different types of exercises for the knee osteoarthritis, and a combination of open and closed isotonic exercises are recommended knee osteoarthritis (26) with the exception to those individuals who find these exercises difficult and painful. In such groups, isometric exercises of knee muscles should be considered (26). In an attempt to manage the knee osteoarthritis, one can exacerbate the symptoms of knee arthritis by following ineffective or unsafe exercises leading to poor prognosis and poor adherence to these exercises (27), hence one should choose the exercises carefully.

As knee arthritis is a significant issue in the elderly population, therefore, the less expensive community-based approach will be beneficial for these patients. There are previous studies which have assessed the efficacy of home-based exercises and reported good results (12, 14, 28) however there are only a few studies which looked at the web-based exercises on the knee arthritis (29-33). Unfortunately, most of these studies recruited patients with knee pain, and radiographic evidence of knee osteoarthritis was undermined except one study (31). It infers that these web-based exercises can be used for knee pain which may include cases of soft tissue injuries, arthritic knee or any other reason for knee pain. However, generalising these results on the group of patients with knee osteoarthritis warrants a risk of increasing their pain just in case that those exercises are strenuous or more demanding.

The aim of this study is to explore the benefits of web-based exercises in the patients with knee osteoarthritis to establish if their pain decreases after six weeks. Being a web-based set of activities, it makes it more accessible to the patients living in rural areas and should make it more cost-effective requiring no travelling time. Our study is different from the other studies as there is not a single study (to our knowledge) which has studied web-based intervention on United Kingdom population of knee osteoartsurgerhritis. Furthermore, we will endeavour to recruit only those individuals who have radiographic evidence of knee osteoarthritis. Therefore, this study will produce recommendations for the clinical setups where most of the symptomatic patients have radiographic evidence of knee osteoarthritis. We will be using a web-based exercises platform known as Joint Academy (JA) as recent pilot studies (32, 33) demonstrated promising results for this platform. This programme is based on Swedish face to face self-management program known as ‘Artrosskolan’ (*The Osteoarthritis School*) which provides structured information and exercises for knee arthritis to the relevant population suffering from knee arthritis. The efficacy of it is covered elsewhere (34). The company that produced the Joint Academy platform has given consent to use their web based platform to conduct this study.

We will recruit 134 individuals with the clinical evidence of radiographic knee arthritis, and these will be randomised into a control group and an interventional group (67 individuals in each group). The interventional group will get the login details for the Joint Academy (JA), and

they will follow the strengthening exercises for six weeks with educational material online relevant to knee osteoarthritis. They will record their pain levels on the Numerical rating scale (NRS) at baseline and at the end of the trial. The validity and sensitivity of NRS have been established numerous times (35-39). Other than determining the subjective pain levels, we will use standardised quantitative sensory testing (QST) such as pressure pain threshold (PPT), temporal summation (TS) and conditioned pain modulation (CPM). Pressure Pain threshold (PPT) has been used in previous studies done on the knee arthritis and deemed to be a valid and reliable method to establish tenderness around the knee joint (40-45). Similarly, temporal summation (TS) has been used in previous studies (40, 43, 44, 46) to establish whether individuals have an element of central sensitisation thus amplifying their localised knee pains. Conditioned pain paradigms are commonly used to assess the function of endogenous pain inhibitory pathways in humans. In this technique, a painful test stimulus is evaluated in the absence and in the presence of a second painful (conditioning) stimulus applied to a remote region of the body. In a typically functioning nociceptive system, the amount of pain experienced with the primary test stimulus will be reduced during the presentation of the secondary conditioning stimulus (47-49). Decreased inhibition of experimental pain is found in many patients with idiopathic pain syndromes (50-58). It predicts the tendency to develop future chronic pain (59, 60). The purpose of using QST is to establish objective data as to whether these exercises reduce the localised tenderness or global central sensitisation.

We will use the Western Ontario and McMaster Universities Osteoarthritis Index (WOMAC) which is widely utilised in the evaluation of Hip and Knee Osteoarthritis. It is a self-administered questionnaire made of 24 items and consists of three subscales covering pain, stiffness and physical function. It has been used extensively and deemed to be a valid and reliable tool (61-65). For the quality of life, stiffness, generalised well-being, difficulty with sleeping and understanding of the diagnosis and treatment, we will use patient-reported outcome measure 'The Arthritis Research UK Musculoskeletal Health Questionnaire' (MSK-HQ). MSK-HQ covers a broad aspect of musculoskeletal conditions, and recent studies have shown it to be reliable and valid (66, 67).

Both the 30-second sit to stand test (30CST) and the 'time up and go' (TUG) test will be used to see if patients have improved their lower limb fitness levels. 30CST has shown excellent reliability and validity (65, 68, 69). TUG has been widely used in clinical setups and is a valid tool to assess necessary functional mobility (70-76).

In this study, we aim to assess the sleeping pattern of individuals suffering from degenerative changes of the knee and purpose is to determine whether sleeping patterns improve when the exercises for knee osteoarthritis are introduced. Disturbed sleep is a frequent complaint of people experiencing chronic pain such as those with knee osteoarthritis (OA) (77-83). The resultant changes in sleep architecture can affect health even in the presence of apparently adequate sleep duration. For example, an insufficient amount of slow wave sleep associates with hypertension, type 2 diabetes mellitus, poor cognition and obesity (84-89).Sleep disturbances are present in 67–88% of people with chronic pain and ≥50% individuals with insomnia have chronic pain (85, 90). Actigraphy will be used to assess the sleeping data which has been used widely and deemed as a valid method (91-96).We will also use Pittsburgh sleep quality index (PSQI) which has been used in multiple studies and validated to measure sleep disturbances (97-101).

We will also endeavour to assess the isokinetic strength of quadriceps to establish if these exercises improve the dynamic strength of knee extensors. Quadriceps muscles strength deficits are associated with knee osteoarthritis (102). Isometric testing will be done at 60 and 180 degrees of flexion as done in a previous study (103).

We will also extract the blood samples at baseline and after six weeks to assess the biomarkers and to establish insulin resistance (104, 105). A urine sample will be taken as well.

Studying these parameters will help us to understand the traits of osteoarthritis, their correlation to each other and potential detrimental effects of them on the health of knee joint. This study will help us to establish whether web-based intervention can replicate the results which it has produced in Swedish population and if it does, then next target will be to assess the socio-economic benefits of this programme over the standard treatment for knee arthritis provided in the community set up in the United Kingdom.

All the methods which have been mentioned above are safe, and procedures have been done previously safely at Academic Rheumatology.

STUDY OBJECTIVES AND PURPOSE

PURPOSE

The purpose of this study is to establish the benefits of web-based exercises in the community-based participants with knee osteoarthritis to see if their pain decreases after six weeks. This will be undertaken primarily by randomised controlled trial in a hospital setup.

PRIMARY OBJECTIVE

1. Carry out an exercise intervention in the individuals with osteoarthritis pain to assess the effect on sleep, central and peripheral pain measures

SECONDARY OBJECTIVES

1. To link the changes in pain, sleep and physical activity to muscle mass, biomarkers of insulin resistance, inflammation and pain.
2. To test individualised web (or app) based delivery of exercise intervention designed specifically for OA pain in the United Kingdom population. The key advantages of the system are costs savings for the health provider and increased long-term compliance.

DETAILS OF PRODUCTS

We will be using web-based exercises platform known as Joint Academy (JA), and recent pilot studies (32, 33) demonstrated promising results. This programme is based on Swedish face to face self-management program known as 'Artrosskolan' (*The Osteoarthritis School*) which provides structured information and exercises for knee arthritis to the relevant population suffering from knee arthritis. The efficacy of it is covered elsewhere (34).

Description

Joint Academy Exercises

Manufacture

Arthro
Stortorget 31, 211 32 Malmö, Sweden
This web based platform and app is CE marked.

Known Side Effects

Increased knee, hip or low back pain and one reported case of increased hypertension (106).

STUDY DESIGN

A multi-centre randomised controlled trial in primary care setting

Study Configuration

A single-centred randomised controlled trial in the primary care setting with participants suffering from the knee arthritis, 1:1 randomised to web based exercises or usual care.

Randomization and Blinding

Participants will undergo computer-generated stratified randomisation by a statistician at Academic Rheumatology using the https://sealedenvelope.com/ software. Generation of a randomisation schedule will include obtaining the random numbers and assigning random numbers to each subject under the specific treatment conditions. Participants will be equally allocated between treatment arms with at least n=67 per arm.

Masking: Not applicable, the study is not double-blinded as participants will find out their allocated group, however, the examiner will be blinded to the intervention.

Maintenance of Randomisation Codes and Procedures for Breaking Code

As per cohort multiple randomised controlled trial design mentioned by another study (107), individuals will have an assessment to establish their suitability for this study and once, that has been established we will randomise them in to control group and interventional group. At this occasion, individuals will find out that they belong to one group or the other and hence blinding is not possible.

As the online system will be used for randomisation, therefore, code break will be kept online, and the research manager will be responsible for keeping a track if it.

Study Management

The study will be managed in Academic Rheumatology, Clinical Sciences Building by the Research Manager under the supervision of the Chief Investigator who has overall responsibility for the study and shall oversee all study management.

The study management group will meet regularly and have documented quarterly meetings from the start of the study. The group will also monitor and supervise the progress of the study and advice on recruitment rate and unforeseen logistical issues to ensure that its objectives are achieved.

The data custodian will be CI who will also have overall responsibility for the study and shall oversee all study management.

Duration of the Study and Participant Involvement

Study Duration and Schedule of Events:

The trial is expected to start in spring 2018 (May – June 2018), and enrolment will end in in the late summer of 2020. After about two months from the end of recruitment, all data will be collected. Data analysis will be performed by a PhD student supervised by CI.

Participant Duration:

Six- Eight weeks including any induction period.

End of the Trial

The end of the trial will be on the last participant visit.

Selection and Withdrawal of Participants

Recruitment

A selection of eligible people for the study will be invited from existing databases held at Academic Rheumatology of participants with knee pain who have agreed to be contacted for future studies. Any shortfall in the recruitment will be compensated by sending study leaflet to GP surgeries. Potentially eligible people are all men and women, aged between 45 years and older, registered with the GP surgery. The screening of existing records can be done by members of their direct healthcare team for those who had x-rays and osteoarthritis of the knee had been established. The GPs will exclude anyone with a known terminal illness, severe psychiatric illness or dementia, or with any other condition or circumstance that makes them unsuitable for the study. All those individuals who are suitable for the study will be sent the study information sheet so they can have some time to think about the research and what is involved in this study.

The study team in Academic Rheumatology will invite interested participants to start the study and this will be considered as the first session. The purpose of the visit will be to establish suitability by proceeding with radiographs for those who have not had x-rays in last 12 months. Those who had x-rays in previous 12 months and qualified for this study will be an exception from x-rays, and they would proceed with the assessment after signing the consent form. The research team will seek consent from the individuals who have not had X- rays, and they will go through the x-rays of a painful knee. If their x-rays demonstrate radiographic evidence of osteoarthritis (K/L= 1 or above), will be included in the study. Those individuals, where their K/L score <1, will be considered as screening failure and will be excluded from the study. All qualifying individuals will be randomised to interventional and control group.

Participants will be free to leave the study at any time without giving any reason. Participants who indicate in their questionnaire that they are willing to receive information about future OA studies may withdraw this indication at any time, and this will be altered appropriately on the

study database. Participants will be made aware via the questionnaire that all data will remain confidential and will be anonymised before use in further analysis.

It will be explained to the potential participant that entry into the trial is entirely voluntary and that their treatment and care will not be affected by their decision. It will also be explained that they can withdraw at any time, but attempts will be made to avoid this occurrence. In the event of their withdrawal, it will be explained that their data collected so far cannot be erased, and we will seek consent to use the data in the final analyses where appropriate (via the information sheet and consent form). Those who withdraw from the study will be made aware that this will not in any way affect their future care.

Inclusion criteria: Aged 45 years and onward.
Clinical diagnosis of knee arthritis with complaints of knee pain for 3 or more months, early morning stiffness <30 minutes, crepitus, bony tenderness, and no palpable warmth and radiographically established osteoarthritis (at least score 1 on K/L scale)
Able to read and write English
Able to use/access computer or tablet and have access to internet

Exclusion criteria: Inability to give informed consent – (capacity levels are already established under GP care)
Terminal or mental illness
Neurological conditions, inflammatory joint diseases including rheumatoid arthritis, gout or calcium pyrophosphate deposition disease (CPPD), and dementia
Participants with sleep apnea
Acute soft tissue injury to the knee within last 3 months before recruiting

Unstable heart condition or rapid fluctuations in hypertension
BMI>50

Informed consent

All participants will provide written informed consent. The Informed Consent Form will be signed and dated by the participant before they enter the trial. The Investigator will explain the details of the trial and provide a Participant Information Sheet, ensuring that the participant has sufficient time to consider participating or not. The Investigator will answer any questions that the participant has concerning study participation.

Informed consent will be collected from each participant before they undergo any interventions (including physical examination and history taking) related to the study. One copy of this will be kept by the participant, one will be kept by the Investigator, and a third will be retained in the participant's hospital records.

Should there be any subsequent amendment to the final protocol, which might affect a participant's participation in the study at any part, continuing consent will be obtained using an amended Consent form which will be signed by the participant.

Future contact

Participants will be asked to indicate on the questionnaire whether they are willing for their contact details to be stored on a secure database in Academic Rheumatology, to enable them to be informed of future studies relating to knee pain and OA undertaken by Academic Rheumatology and the Pain Centre for which they might be suitable. Participant data will be kept up-to-date in accordance with Data Protection Act, 1998. The consent form includes an

optional clause asking participants to confirm whether they are happy for their personal details to be retained for this purpose - if not their contact details will be destroyed at the end of this study after it is no longer necessary to contact them.

Study Regimen

Participants seeking care for osteoarthritis will be informed of the study both orally and in writing. Those who qualify for the study will sign the informed consent and the participants will be randomised to interventional group and control group. The exception applies to those Participants who have not had knee X-rays in last 12 months. These individuals will be called to the hospital. After gaining a valid consent, knee radiographs will be taken, and their X-rays will be verified by experienced staff. Once, a definite eligibility criterion (K/L score at least 1 or above) is established in such cases, the potential participants will be randomised to interventional group and control group like the other individuals. Blinding such interventional studies are difficult to achieve as participants will establish as to in which group they are in however study will be blinded to the examining clinician. Controlled and interventional groups individuals will be recruited for this study. Interventional groups will have an assessment session with the experienced staff and NRS, QST, WOMAC, MSK-HQ, 30CST, TUG, PSQI, MSK-USS, urine and blood samples will be taken at baseline.

Interventional group will shortly after that receive a link via email, which will be used to log-in to Joint Academy online portal. After log-in has been achieved, the intervention starts. It consist of a 6-week internet-based physical therapy program that provides information, exercises, contact details of a personal physiotherapist, education about lifestyle and behavioural changes. This intervention can be accessed using a smartphone, a tablet or a computer. The program encourages the physical activity by sending email prompts on a regular basis to help participants to continue during the whole treatment. Initially, participants will answer an online-questionnaire covering areas such as joint pain intensity, health-related quality of life, physical function as well as being instructed in and performing a physical test assessing lower limb strength / physical function. This questionnaire will form a part of online baseline assessment. Exercises itself consistent of knee and hip exercises along with some functional activities such as sit to stand and stairs climbing. Interventional group will be given actigraphy device (a device to monitor sleeping pattern) which is CE marked. Therefore, their sleeping pattern can be recorded quantitatively.

Once exercises programme is finished in six weeks, the participants will fill in the same questionnaire and perform the physical tests, to enable evaluation. There are two face-to-face meetings between participants and physiotherapist/nurse, at enrolment and after six weeks. The physiotherapist is also available via asynchronous online chat or over the phone during the 6-week study period. After six weeks, participants can continue with sustain-program for self-management, mainly focusing on the continuation of treatment, performing the exercises regularly.

Control group will continue with their routine self-management which is offered in the community setup. They will be assessed on NRS, QST, WOMAC, MSK-HQ, PSQI, 30CST, TUG, isokinetic muscles strengthen of quadriceps, urine and blood samples at baseline. Control group will also get the actigraphy to monitor sleeping pattern of that group. They will be re-assessed after six weeks on the primary objective measures to see if they have made any difference by following self-management strategies in the community.

The suggested study utilises a pragmatic approach, where all participants with clinical knee OA that would have been eligible for a face-to-face program are included if they can handle a computer, tablet or smartphone.

To summarise, the total study duration, including recruitment and the two assessment visits, will be 12-15 months. The key stages are detailed below;

- Individuals who agree to attend for a clinical assessment will participate for two appointments. This appointment will last approximately 2-3 hours.
- They will be seen at the Academic Rheumatology Unit, Clinical Sciences Building, Nottingham City Hospital and will be asked to come between 8.00am and 10.00am without having had breakfast or any drink other than water.
- Transport (taxi) will be arranged if required and any travel expense will be reimbursed.
- Our Research Nurses will get a 15ml blood sample from the participant and also ask the participant to provide a urine sample.
- Once blood and urine samples have been collected participants will be offered a light breakfast and drink refreshment before a brief assessment to confirm medical history and current medications. Questionnaires such as WOMAC, MSK-HQ and Numeric Rating Scale will be collected.
- Physiological measurements such as 30 seconds sit to stand (30CST), time up and go (TUG) and Muscle strength assessment will be taken at this point.
- Quantitative sensory testing to test the sensitivity to mechanical pressure and pain.
- Participants will be asked if they wish to donate a faecal sample and if they agree they will be given a collection kit to take home, which will be shipped back by post.
- Participants will be shown how to wear actigraphy device and will be familiarised with the equipment. Each participant will take one device with them, therefore, their sleeping pattern can be assessed.
- Participant will be expected to login to online exercises portal on daily basis and complete the exercises which consist of 30 minutes of exercises and some educational videos.

Muscle and Strength Measurements

A sequence of three physiological measures will be undertaken:

Time up and Go: The participant will start in a seated position.
The participant will stand up upon therapist’s command, walk 3 meters, turn around, walk back to the chair and sits down.
The time will stop when the participant is seated.
The subject can use an assistive device. If the assistive device is used, it will be documented.
NOTE: A practice trial will be completed before the timed trial

***30 seconds sit to stand*.** The 30-Second Chair Test is administered using a folding chair without arms, with a seat height of 17 inches (43.2 cm). The chair, with rubber tips on the legs, is placed against a wall to prevent it from moving.
The participant is seated in the middle of the chair, back straight; feet approximately shoulder width apart and placed on the floor at an angle slightly back from the knees, with one foot

slightly in front of the other to help maintain balance. Arms are crossed at the wrists and held against the chest.
Demonstrate the task both slowly and quickly.
Have the participant practice a repetition or 2 before completing the test.
If a participant must use their arms to complete the test, they are scored 0.
The participant is encouraged to complete as many full stands as possible within 30 seconds.
The participant is instructed to fully sit between each stand.
While monitoring the participant's performance to ensure proper form, the tester silently counts the completion of each correct stand. The score is the total number of stands within 30 seconds (more than halfway up at the end of 30 seconds counts as a full stand). Incorrectly executed stands are not counted.
The 30-second chair stand involves recording the number of stands a person can complete in 30 seconds rather than the amount of time it takes to complete a pre-determined number of repetitions.

Muscle strength Assessment: Isokinetic testing will be done at 60 and 180 degrees of flexion as done in previous study (103) and participant will be in sitting position with hips and knees strapped to keep the position standardised.

Pressure Pain threshold (PPT)

PPT is a non-invasive test during which the sensitivities of the nerves are assessed by recording the smallest force applied to the skin. When this force by the surface area of the skin is applied (pressure), this will be felt as mild, temporary pain and recorded. The pressure probe used consists of a rod with an end the size of a 5p piece, mounted in a handheld device connected to a computer. The force with which the probe is pressed onto the skin is gradually increased until the participant indicates (by pressing a button) that the sensation has changed from pressure to pain. The probe is then automatically immediately taken off the skin. The probe will be used on knees, and proximal shins using a standardised protocol used in other studies within the Pain Centre. Each region will be tested three times with short rest periods between. The participants will be familiarised with the test before it is administered so that they know what to expect and how to respond.

Continuous Pain Modulation (CPM)

CPM will be done along with PPT testing. The reference point for PPT testing will be 3cm medial to patella on the most painful knee. Verbal instructions will be given to the participant. The Numerical Rating Scale (NRS) target will be ≥4 out of 10 from the cuff pressure. Staff/ clinician will wrap 7.5cm wide tourniquet cuff around the contralateral arm to the knee being tested. The lower rim of tourniquet cuff will be kept 3cm proximal to cubital fossa. Systolic pressure will be set to 270mmHg. After target pressure is achieved, the participant will be asked to rate sensation in the arm from 0-10. The participant will be asked to make hand grip ≥10 times until NRS of 4 will be reached. NRS rating will be asked every five hand grips. Once NRS of 4 will be achieved, the probe of algometer will be applied in the same manner as before to knee test site (during PPT testing). Once the participant presses the button, the probe will be withdrawn, and cuff will be released from the elbow. Participants will be advised to wait until cuff evoked pain subsides before re-test, and a minimum of 1 minute should be spared. PPT test will be repeated again (without the cuff now). Their difference in PPT score (with conditioning – without conditioning) will establish the CPM effects. Positive value predicts efficient and negative value predicts in-effective CPM.

Temporal Summation (TS)

The mechanical temporal summation is a non-invasive test during which repetitive mechanical stimulation is applied over a short period to get their augmented response. Increased pain response to a repeated mechanical stimulus may indicate enhanced central sensitisation. The test site will be a suprapatellar region (5 cm proximal from the central part of patella).
A 256mN weighted pinprick stimulator will be used and applied perpendicular to the skin of suprapatellar region of affected knee (5cm proximal from the centre of patella). The participant

will be asked to rate the pain or sharpness they experience from 0-100 where 0 indicates no pain or sharpness and 100 indicates the most intense pain or sharpness imaginable. Numerical Rating Scale (NRS) with verbal descriptors will be used. The response of the participant will be recorded. The same stimulator at the same site will be applied ten times repeatedly at a rate of 1/second. The size of the site would be kept approximately 1 cm square. At the end of the series of 10 pinpricks, the participant will be asked to rate the pain or sharpness which they experience averaged over the whole series of 10 stimuli using the same NRS. The mechanical temporal summation reading will be calculated as the difference between two ratings which is second rating minus the first rating.

Compliance

Compliance will be judged by the online participation of individuals to follow their exercises and by the attendance of two face to face sessions. E-mail prompts are routinely sent out reminding individuals of their exercises. Furthermore, individuals will be given a contact number to contact us as to if they have any query.

Criteria for terminating the study

As the study involves two assessments only and does not involve investigational medicinal products or medical devices it is not envisaged that circumstances will arise that require termination of this study.

Radiographic Evaluation

Tibiofemoral and patellofemoral radiographs will be taken using a standardised protocol (standing posterior-anterior (PA) and skyline views) and will be scored by a single experienced observer. A Perspex Rosenberg template with lead beads is used for the standing PA view to standardising the degree of knee flexion, foot rotation and magnification (108). PA radiographs are taken with the participant facing the x-ray tube while standing on the Rosenberg jig and leaning forwards with their thighs touching the anterior aspect of the jig, the x-ray beams passing from the posterior aspect through to the anterior aspect of the knee. Variable jigs are used for the skyline view to obtaining 30^0 of knee flexion with the participant lying in a reclined supine position on a couch

Grading of radiographs for changes of OA will be based on the Kellgren and Lawrence (K/L) score (109, 110).

Radiation Exposure

This study involves exposure to a very small amount of additional radiation. As part of everyday living, everyone is exposed to naturally occurring background radiation. The effective dose for this study has been estimated to be roughly equivalent to half a day of average background radiation in the UK. At this dose level, no harmful effects of radiation have been demonstrated as any effect would be too small to measure. The risk is believed to be minimal.

The following effective dose figures have been estimated using PCXMC 2.0 and either exposure factors & radiographic technique or audit data on DAPs/examination.

- Semi-flexed weight-bearing view 0.62μSv
- Skyline view (both knees) 1.0 μSv (One knee) 0.5 μSv

If a participant received the highest doses, then this would give ≈1.6μSV for all exposures. Bearing in mind that practice varies between sites, other dose data suggests that doses may vary by a factor of two. Therefore we could assume a maximum of 3μSV per participant from research exposures.

Transport and Storage of the Tissues

The sample will be stored at the premises which belongs to University of Nottingham. Whole blood (5ml) samples will be stored in a refrigerator (at -20°C). Remaining blood (10ml) will be centrifuged as soon after venesection as possible and the serum stored, with the urine, at -80C within freezers in University of Nottingham premises at the Clinical Sciences Building (CSB), City Hospital for future metabolomic studies. Excess samples (serum, urine and faeces) that remain after the study analyses have been undertaken will remain stored within the CSB for possible future research studies related to OA and/or pain provided that participants are agreeable and sign the optional clause on the consent form. These CSB facilities come within the remit of the Research Tissue Bank (DI Dr William Dunn- Licence Number 12265) where participants do not agree to the future use of the samples they will be destroyed in accordance with the Human Tissue Act, 2004.

Samples will be stored in the linked anonymised format in the CSB and labelled using a randomly generated unique participant identifier to permit accurate linkage to clinical data and the consent form. The master database will be held by the CI in an encrypted password file.

Laboratory Analysis

Whole blood may be sent to a specialist company for processing using standard protocols. Bacterial DNA will be extracted from faecal samples to identify bacterial lineages associated with pain and inflammation in OA. Faecal samples will be stored till we have all the samples (as this is optional) and then we will send it to the laboratory. Similarly, biochemical analyses may be undertaken by other academic or commercial groups. Such investigations will be undertaken on anonymised samples under usual Material Transfer Agreement arrangements. Storage of these tissues will be managed, transported and handled according to SOP HTMG 004. Companies which will be used for the analysis should process the samples within seven days upon receipt or companies with HTA licence will be considered.

STATISTICS

Methods

Associate Professor Dr Ana Valdes will oversee the statistical analyses. The data will be entered into MS Access and analysed using SPSS (Statistical Packages for Social Scientists, version 19).

The results of the study will be available to the study management group. All data will be stored in Academic Rheumatology and will be backed up on the University of Nottingham servers.

Sample size and justification

We are anticipating at least 60 individuals in each study arm at the closure of the study.

A recent systematic review of 44 high-quality exercise trials for knee OA pain (3537 participants) (106) found an average effect of 12/100 VAS points corresponding to 0.49 standard deviations. A sample size of n=60 per group is necessary to achieve 75% stat power. The estimated dropout rate for exercise interventions is 12%, therefore, a sample of 67 per arm will be recruited to achieve at least 75% power.

ADVERSE EVENTS

Increase in the knee, hip or back pain are possible adverse effects (106). One case of raised hypertension has been reported by a previous study (111).
Participants will be asked to contact the study site immediately in the event of any serious adverse event. Any adverse event will be recorded and closely monitored until resolution, stabilisation or until it has been shown that the study treatment / intervention is not the cause. Participants can contact physiotherapists (SG) if there is any adverse event. The CI shall be informed immediately of any serious adverse events and shall determine seriousness and causality in conjunction with any treating medical practitioners.

Any serious adverse event (s) related to this study will be recorded and reported to the REC as part of annual reports. Unexpected serious adverse events will be reported within timeframes to the REC and CI will be responsible for all adverse event reporting.

ETHICAL AND REGULATORY ASPECTS

Involvement of Participant's General Practitioner

Participant's GPs will not be informed that their patient has agreed to participate in the study.

Ethics Committee and Regulatory Approvals

The study will not be initiated before the protocol, consent forms and participant information sheets have received approval / favourable opinion from the Research Ethics Committee (REC), and the respective National Health Service (NHS) Research & Development (R&D) department. Should a protocol amendment be made, that requires REC approval, the changes in the protocol will not be instituted until the amendment and revised informed consent forms, and participant and GP information sheets (if appropriate) have been reviewed and received approval / favourable opinion from the REC and R&D departments. A protocol amendment intended to eliminate an apparent immediate hazard to participants may be implemented immediately notifying the REC as soon as possible and approval is requested. Minor protocol amendments only for logistical or administrative changes may be implemented immediately, and the REC will be informed.

The study will be conducted in accordance with the ethical principles that have their origin in the Declaration of Helsinki, 1996; the principles of Good Clinical Practice, and the Department of Health Research Governance Framework for Health and Social Care, 2005.

Informed Consent and Participant Information

The process for obtaining participant informed consent will be in accordance with the REC guidance, and Good Clinical Practice (GCP) and any other regulatory requirements that might be introduced. The investigator or their nominee and the participant shall both sign and date the Consent Form before the person can participate in the study.

The participant will receive a copy of the signed, and dated forms and the original will be retained in the Study records.

The decision regarding participation in the study is entirely voluntary. The investigator or their nominee shall emphasize to them that consent regarding study participation may be withdrawn at any time without penalty or affecting the quality or quantity of their future medical care, or loss of benefits to which the participant is otherwise entitled. No study-specific interventions will be done before informed consent has been obtained.

The investigator will inform the participant of any relevant information that becomes available during the course of the study and will discuss with them, whether they wish to continue with the study. If applicable, they will be asked to sign revised consent forms.

If the Consent Form is amended during the study, the investigator shall follow all applicable regulatory requirements pertaining to approval of the amended Consent Form by the REC and use of the amended form (including for ongoing participants).

Records

Study Forms

Each participant will be assigned a study identity code number, and this will be used on the study forms as well as other trial documents and the electronic database. This will be a randomly generated unique participant identifier. An additional identifier will be sued to allow validation of participants ID and prevent number being transposed. This additional identifier will consistent of participant's initials at the end of unique participant identifier number.

Study forms will be treated as confidential documents and held securely in accordance with regulations. The investigator will make a separate confidential record of the participant's name, date of birth, local hospital number or NHS number, and Participant Study Number, to permit identification of all participants enrolled in the trial, in accordance with regulatory requirements and for follow-up as required.

Study forms shall be restricted to those personnel approved by the Chief or local Principal Investigator and recorded in a Study Personnel Log. All paper forms shall be filled in using a black ballpoint pen. Errors shall be lined out but not obliterated by using correction fluid and the correction inserted, initialled and dated.

The Chief or local Principal Investigator shall sign a declaration ensuring the accuracy of data recorded in the study form.

Sample Labelling

Each participant will be assigned a unique study identification code number for use on the samples, consent forms and other study documents and the electronic database.

Source documents

Source documents shall be filed at the investigator's site and may include but are not limited to, consent forms, study records, field notes, interview transcriptions and audio records. A study form may also completely serve as its own source data.

Direct access to source data/documents

The study form and all source documents, including progress notes and copies of laboratory and medical test results, shall be made be available at all times for review by the Chief Investigator, Sponsor's designee and inspection by relevant regulatory authorities.

Data Protection

All trial staff and investigators will endeavour to protect the rights of the trial's participants to privacy and informed consent and will adhere to the Data Protection Act, 1998. The study forms will only collect the minimum required information for the purposes of the trial. Study forms will be held securely, in a locked room, or locked cupboard or cabinet. Access to the information will be limited to the trial staff and investigators and relevant regulatory authorities (see above). The computer held data including the trial database will be held securely and password protected. All data will be stored on a secure dedicated web server. Access will be restricted by user identifiers and passwords (encrypted using a one-way encryption method). Information about the trial in the participant's medical records / hospital notes will be treated confidentially in the same way as all other confidential medical information.

Electronic data will be backed up every 24 hours to both local and remote media in encrypted format

QUALITY ASSURANCE & AUDIT

Insurance and Indemnity

Insurance and indemnity for clinical study participants and study staff are covered within the NHS Indemnity Arrangements for clinical negligence claims in the NHS, issued under cover of HSG (96)48. There are no special compensation arrangements, but study participants may have recourse through the NHS complaints procedures.

The University of Nottingham as research sponsor indemnifies its staff, research participants and research protocols with both public liability insurance and clinical trials insurance. These policies include provision for indemnity in the event of a successful litigious claim for proven non-negligent harm.

Study Conduct

Study conduct will be subject to systems audit for inclusion of essential documents; permissions to conduct the study; CVs of study staff and training received; local document control procedures; consent procedures and recruitment logs; adherence to procedures defined in the protocol (e.g. inclusion / exclusion criteria, random selection of suitable participants for Phase II and Phase III, timeliness of visits); accountability of study materials and equipment calibration logs.

Study Data

Monitoring of study data shall include confirmation of informed consent; source data verification; data storage and data transfer procedures; local quality control checks and

procedures, back-up and disaster recovery of any local databases and validation of data manipulation. This will be managed by the direct study team.

Entries on study forms will be verified by inspection against the source data. A sample of the forms (10%) will be checked on a regular basis for verification of all entries made. In addition, the subsequent capture of the data on the study database will be checked. Where corrections are required these will carry a full audit trail and justification.

Study data and evidence of monitoring and systems audits will be made available for inspection by the REC as required.

Record Retention and Archiving

In compliance with the ICH/GCP guidelines, regulations and in accordance with the University of Nottingham Code of Research Conduct and Research Ethics, the Chief or local Principal Investigator will maintain all records and documents regarding the conduct of the study. These will be retained for at least seven years or for longer if required. If the responsible investigator is no longer able to maintain the study records, a second person will be nominated to take over this responsibility.

The study master file held by the Chief Investigator on behalf of the Sponsor shall be finally archived at secure archive facilities at the University of Nottingham. This archive shall include study databases and associated meta-data encryption codes.

Discontinuation of the Trial by the Sponsor

The Sponsor reserves the right to discontinue this study at any time for failure to meet expected enrolment goals, for safety or any other administrative reasons. The Sponsor shall take advice as appropriate in making this decision.

Statement of Confidentiality

Individual participant medical or personal information obtained as a result of this study are considered confidential, and disclosure to third parties is prohibited.

Participant confidentiality will be further ensured by utilising identification code numbers to correspond to treatment data in the computer files.

Data generated as a result of this study will be available for inspection on request by the participating physicians, the University of Nottingham representatives, the REC, local R&D Departments and the regulatory authorities.

PUBLICATION AND DISSEMINATION POLICY

The study results will be submitted to Arthritis Research UK, regulatory authorities, and peer-reviewed journals for publication. In addition, the results will be presented at national and international conferences. Study participants' identity will not be disclosed when publishing the results. Study participants will also be informed of the results if requested.

USER AND PUBLIC INVOLVEMENT

On the PPI representative meeting of 14/12/2015, seven volunteers suffering from chronic arthritis pain were asked about the relevance of studying sleep in the context of OA pain. They all were very supportive of studying and understanding sleep patterns. They viewed

actigraphy as a good non-invasive alternative to polysomnography and saw no problem with using the device for 6 weeks.
On 17/05/2017 6 representatives from the PPI MSK group were asked about exercise interventions and were all supportive of this. They were also asked about the extraction of synovial fluid from their joints. Five out of six said they would not have a problem with this if it was performed by a specialist using ultrasound to guide the needle, which is why this is an optional part of the protocol.
The latest versions of the participant facing documentation (PIS, consent form, invitation letter and flyer) has all been forwarded to three PPI representatives who have reviewed and commented on it.

STUDY FINANCES

Funding source

This study is part-funded by the Arthritis Research UK Pain Centre (University of Nottingham) and part-funded by MRC Arthritis Research UK Centre for MSK Ageing Research, School of Life Sciences, University of Nottingham.

Participant stipends and payments

Participants will not be paid to participate in the study. However, all travel expenses to and from Academic Rheumatology, Clinical Sciences Building, Nottingham City Hospital will be covered.

SIGNATURE PAGES

Signatories to Protocol:

Chief Investigator: (name)________________________________

Signature:________________________________

Date: ___________

Co- investigator: (name) ________________________________

Signature:________________________________

Date: ___________

Trial Statistician: (name)________________________________

Signature:________________________________

Date: ___________

REFERENCES

1. Peat G, McCarney R, Croft P. Knee pain and osteoarthritis in older adults: a review of community burden and current use of primary health care. Ann Rheum Dis. 2001;60(2):91-7.
2. Felson DT. Weight and osteoarthritis. The American journal of clinical nutrition. 1996;63(3 Suppl):430s-2s.
3. Guccione AA, Felson DT, Anderson JJ, Anthony JM, Zhang Y, Wilson PW, et al. The effects of specific medical conditions on the functional limitations of elders in the Framingham Study. American Journal of Public Health. 1994;84(3):351-8.
4. Royal College of General P, McCormick A, Fleming D, Charlton J, Great B, Office of Population C, et al. Morbidity statistics from general practice : fourth national study 1991-1992. London: H.M.S.O.; 1995.
5. Conaghan PG, Dickson J, Grant RL. Care and management of osteoarthritis in adults: summary of NICE guidance. BMJ : British Medical Journal. 2008;336(7642):502-3.
6. Jevsevar DS. Treatment of osteoarthritis of the knee: evidence-based guideline, 2nd edition. The Journal of the American Academy of Orthopaedic Surgeons. 2013;21(9):571-6.
7. Pendleton A, Arden N, Dougados M, Doherty M, Bannwarth B, Bijlsma JW, et al. EULAR recommendations for the management of knee osteoarthritis: report of a task force of the Standing Committee for International Clinical Studies Including Therapeutic Trials (ESCISIT). Ann Rheum Dis. 2000;59(12):936-44.
8. Bennell KL, Hunter DJ, Hinman RS. Management of osteoarthritis of the knee. BMJ (Clinical research ed). 2012;345:e4934.
9. Hochberg MC, Altman RD, April KT, Benkhalti M, Guyatt G, McGowan J, et al. American College of Rheumatology 2012 recommendations for the use of nonpharmacologic and pharmacologic therapies in osteoarthritis of the hand, hip, and knee. Arthritis Care Res (Hoboken). 2012;64(4):465-74.
10. Bennell KL, Hinman RS. A review of the clinical evidence for exercise in osteoarthritis of the hip and knee. Journal of science and medicine in sport. 2011;14(1):4-9.
11. Messier SP, Loeser RF, Miller GD, Morgan TM, Rejeski WJ, Sevick MA, et al. Exercise and dietary weight loss in overweight and obese older adults with knee osteoarthritis: The arthritis, diet, and activity promotion trial. Arthritis & Rheumatism. 2004;50(5):1501-10.
12. Thomas KS, Muir KR, Doherty M, Jones AC, O'Reilly SC, Bassey EJ. Home based exercise programme for knee pain and knee osteoarthritis: randomised controlled trial. BMJ (Clinical research ed). 2002;325(7367):752.
13. van Baar ME, Assendelft WJ, Dekker J, Oostendorp RA, Bijlsma JW. Effectiveness of exercise therapy in patients with osteoarthritis of the hip or knee: a systematic review of randomized clinical trials. Arthritis and rheumatism. 1999;42(7):1361-9.
14. Chamberlain MA, Care G, Harfield B. Physiotherapy in osteoarthrosis of the knees. A controlled trial of hospital versus home exercises. International rehabilitation medicine. 1982;4(2):101-6.
15. Ettinger WH, Jr., Burns R, Messier SP, Applegate W, Rejeski WJ, Morgan T, et al. A randomized trial comparing aerobic exercise and resistance exercise with a health education program in older adults with knee osteoarthritis. The Fitness Arthritis and Seniors Trial (FAST). Jama. 1997;277(1):25-31.
16. Ettinger WH, Jr, Burns R, Messier SP, et al. A randomized trial comparing aerobic exercise and resistance exercise with a health education program in older adults with knee osteoarthritis: The fitness arthritis and seniors trial (fast). Jama. 1997;277(1):25-31.
17. Kovar PA, Allegrante JP, MacKenzie CR, Peterson MG, Gutin B, Charlson ME. Supervised fitness walking in patients with osteoarthritis of the knee. A randomized, controlled trial. Annals of internal medicine. 1992;116(7):529-34.

18. Minor MA, Hewett JE, Webel RR, Anderson SK, Kay DR. Efficacy of physical conditioning exercise in patients with rheumatoid arthritis and osteoarthritis. Arthritis and rheumatism. 1989;32(11):1396-405.
19. Fisher NM, Pendergast DR, Gresham GE, Calkins E. Muscle rehabilitation: its effect on muscular and functional performance of patients with knee osteoarthritis. Archives of physical medicine and rehabilitation. 1991;72(6):367-74.
20. Pisters MF, Veenhof C, van Meeteren NLU, Ostelo RW, de Bakker DH, Schellevis FG, et al. Long-Term effectiveness of exercise therapy in patients with osteoarthritis of the hip or knee: A systematic review. Arthritis Care & Research. 2007;57(7):1245-53.
21. Hurley MV, Walsh NE, Mitchell HL, Pimm TJ, Patel A, Williamson E, et al. Clinical effectiveness of a rehabilitation program integrating exercise, self-management, and active coping strategies for chronic knee pain: A cluster randomized trial. Arthritis Care & Research. 2007;57(7):1211-9.
22. Dunlop DD, Song J, Semanik PA, Sharma L, Chang RW. Physical activity levels and functional performance in the osteoarthritis initiative: a graded relationship. Arthritis and rheumatism. 2011;63(1):127-36.
23. Esser S, Bailey A. Effects of exercise and physical activity on knee osteoarthritis. Current pain and headache reports. 2011;15(6):423-30.
24. Lange AK, Vanwanseele B, Fiatarone Singh MA. Strength training for treatment of osteoarthritis of the knee: a systematic review. Arthritis and rheumatism. 2008;59(10):1488-94.
25. Mikesky AE, Mazzuca SA, Brandt KD, Perkins SM, Damush T, Lane KA. Effects of strength training on the incidence and progression of knee osteoarthritis. Arthritis Care & Research. 2006;55(5):690-9.
26. Baker K, McAlindon T. Exercise for knee osteoarthritis. Current opinion in rheumatology. 2000;12(5):456-63.
27. Peeler J, Ripat J. The effect of low-load exercise on joint pain, function, and activities of daily living in patients with knee osteoarthritis. The Knee. 2018.
28. Talbot LA, Gaines JM, Huynh TN, Metter EJ. A Home-Based Pedometer-Driven Walking Program to Increase Physical Activity in Older Adults with Osteoarthritis of the Knee: A Preliminary Study. Journal of the American Geriatrics Society. 2003;51(3):387-92.
29. Lorig KR, Ritter PL, Laurent DD, Plant K. The internet-based arthritis self-management program: A one-year randomized trial for patients with arthritis or fibromyalgia. Arthritis Care & Research. 2008;59(7):1009-17.
30. Bossen D, Veenhof C, Van Beek KEC, Spreeuwenberg PMM, Dekker J, De Bakker DH. Effectiveness of a Web-Based Physical Activity Intervention in Patients With Knee and/or Hip Osteoarthritis: Randomized Controlled Trial. Journal of Medical Internet Research. 2013;15(11):e257.
31. Brooks MA, Beaulieu JE, Severson HH, Wille CM, Cooper D, Gau JM, et al. Web-based therapeutic exercise resource center as a treatment for knee osteoarthritis: a prospective cohort pilot study. BMC musculoskeletal disorders. 2014;15:158.
32. Dahlberg LE, Grahn D, Dahlberg JE, Thorstensson CA. A Web-Based Platform for Patients With Osteoarthritis of the Hip and Knee: A Pilot Study. JMIR research protocols. 2016;5(2):e115.
33. Nero H, Dahlberg J, Dahlberg EL. A 6-Week Web-Based Osteoarthritis Treatment Program: Observational Quasi-Experimental Study. J Med Internet Res. 2017;19(12):e422.
34. Thorstensson CA, Garellick G, Rystedt H, Dahlberg LE. Better Management of Patients with Osteoarthritis: Development and Nationwide Implementation of an Evidence-Based Supported Osteoarthritis Self-Management Programme. Musculoskeletal care. 2015;13(2):67-75.
35. Ferreira-Valente MA, Pais-Ribeiro JL, Jensen MP. Validity of four pain intensity rating scales. PAIN®. 2011;152(10):2399-404.

36. Hjermstad MJ, Fayers PM, Haugen DF, Caraceni A, Hanks GW, Loge JH, et al. Studies Comparing Numerical Rating Scales, Verbal Rating Scales, and Visual Analogue Scales for Assessment of Pain Intensity in Adults: A Systematic Literature Review. Journal of Pain and Symptom Management. 2011;41(6):1073-93.
37. Karcioglu O, Topacoglu H, Dikme O, Dikme O. A systematic review of the pain scales in adults: Which to use? The American Journal of Emergency Medicine. 2018.
38. Williamson A, Hoggart B. Pain: a review of three commonly used pain rating scales. Journal of Clinical Nursing. 2005;14(7):798-804.
39. Mawdsley RH, Moran KA, Conniff LA. Reliability of Two Commonly Used Pain Scales With Elderly Patients. Journal of Geriatric Physical Therapy. 2002;25(3):16-20.
40. Arendt-Nielsen L, Nie H, Laursen MB, Laursen BS, Madeleine P, Simonsen OH, et al. Sensitization in patients with painful knee osteoarthritis. Pain. 2010;149(3):573-81.
41. Imamura M, Imamura ST, Kaziyama HH, Targino RA, Hsing WT, de Souza LP, et al. Impact of nervous system hyperalgesia on pain, disability, and quality of life in patients with knee osteoarthritis: a controlled analysis. Arthritis and rheumatism. 2008;59(10):1424-31.
42. Lee YC, Lu B, Bathon JM, Haythornthwaite JA, Smith MT, Page GG, et al. Pain sensitivity and pain reactivity in osteoarthritis. Arthritis Care Res (Hoboken). 2011;63(3):320-7.
43. Neogi T, Frey-Law L, Scholz J, Niu J, Arendt-Nielsen L, Woolf C, et al. Sensitivity and sensitisation in relation to pain severity in knee osteoarthritis: trait or state? Ann Rheum Dis. 2015;74(4):682-8.
44. Neogi T, Guermazi A, Roemer F, Nevitt MC, Scholz J, Arendt-Nielsen L, et al. Association of Joint Inflammation With Pain Sensitization in Knee Osteoarthritis: The Multicenter Osteoarthritis Study. Arthritis & rheumatology (Hoboken, NJ). 2016;68(3):654-61.
45. Wessel J. The reliability and validity of pain threshold measurements in osteoarthritis of the knee. Scand J Rheumatol. 1995;24(4):238-42.
46. Finan PH, Buenaver LF, Bounds SC, Hussain S, Park RJ, Haque UJ, et al. Discordance between pain and radiographic severity in knee osteoarthritis: findings from quantitative sensory testing of central sensitization. Arthritis and rheumatism. 2013;65(2):363-72.
47. Pud D, Granovsky Y, Yarnitsky D. The methodology of experimentally induced diffuse noxious inhibitory control (DNIC)-like effect in humans. PAIN®. 2009;144(1):16-9.
48. Yarnitsky D, Arendt-Nielsen L, Bouhassira D, Edwards RR, Fillingim RB, Granot M, et al. Recommendations on terminology and practice of psychophysical DNIC testing. European Journal of Pain. 2010;14(4):339.
49. Lewis GN, Rice DA, McNair PJ. Conditioned Pain Modulation in Populations With Chronic Pain: A Systematic Review and Meta-Analysis. The Journal of Pain. 2012;13(10):936-44.
50. Chang L. Brain Responses to Visceral and Somatic Stimuli in Irritable Bowel Syndrome: a Central Nervous System Disorder? Gastroenterology Clinics of North America. 2005;34(2):271-9.
51. Julien N, Goffaux P, Arsenault P, Marchand S. Widespread pain in fibromyalgia is related to a deficit of endogenous pain inhibition. Pain. 2005;114(1):295-302.
52. Kosek E, Hansson P. Modulatory influence on somatosensory perception from vibration and heterotopic noxious conditioning stimulation (HNCS) in fibromyalgia patients and healthy subjects. Pain. 1997;70(1):41-51.
53. Lautenbacher S, Rollman GB. Possible deficiencies of pain modulation in fibromyalgia. Clinical Journal of Pain. 1997;13(3):189-96.
54. Maixner W, Fillingim R, Booker D, Sigurdsson A. Sensitivity of patients with painful temporomandibular disorders to experimentally evoked pain. Pain. 1995;63(3):341-51.

55. Pielsticker A, Haag G, Zaudig M, Lautenbacher S. Impairment of pain inhibition in chronic tension-type headache. Pain. 2005;118(1):215-23.
56. Rossi P, Serrao M, Perrotta A, Pierelli F, Sandrini G, Nappi G. Neurophysiological approach to central pain modulation in primary headaches. Journal of Headache and Pain. 2005;6(4):191-4.
57. Sandrini G, Rossi P, Milanov I, Serrao M, Cecchini AP, Nappi G. Abnormal modulatory influence of diffuse noxious inhibitory controls in migraine and chronic tension-type headache patients. Cephalalgia. 2006;26(7):782-9.
58. Song GH, Venkatraman V, Ho KY, Chee MWL, Yeoh KG, Wilder-Smith CH. Cortical effects of anticipation and endogenous modulation of visceral pain assessed by functional brain MRI in irritable bowel syndrome patients and healthy controls. Pain. 2006;126(1):79-90.
59. Edwards RR, Ness TJ, Weigent DA, Fillingim RB. Individual differences in diffuse noxious inhibitory controls (DNIC): association with clinical variables. Pain. 2003;106(3):427-37.
60. Yarnitsky D, Crispel Y, Eisenberg E, Granovsky Y, Ben-Nun A, Sprecher E, et al. Prediction of chronic post-operative pain: Pre-operative DNIC testing identifies patients at risk. PAIN. 2008;138(1):22-8.
61. Bellamy N, Buchanan WW, Goldsmith CH, Campbell J, Stitt LW. Validation study of WOMAC: a health status instrument for measuring clinically important patient relevant outcomes to antirheumatic drug therapy in patients with osteoarthritis of the hip or knee. J Rheumatol. 1988;15(12):1833-40.
62. Bombardier C, Melfi CA, Paul J, Green R, Hawker G, Wright J, et al. Comparison of a generic and a disease-specific measure of pain and physical function after knee replacement surgery. Medical care. 1995;33(4 Suppl):As131-44.
63. Hawker G, Melfi C, Paul J, Green R, Bombardier C. Comparison of a generic (SF-36) and a disease specific (WOMAC) (Western Ontario and McMaster Universities Osteoarthritis Index) instrument in the measurement of outcomes after knee replacement surgery. J Rheumatol. 1995;22(6):1193-6.
64. McConnell S, Kolopack P, Davis AM. The Western Ontario and McMaster Universities Osteoarthritis Index (WOMAC): a review of its utility and measurement properties. Arthritis Care & Research. 2001;45(5):453-61.
65. Gill SD, de Morton NA, Mc Burney H. An investigation of the validity of six measures of physical function in people awaiting joint replacement surgery of the hip or knee. Clinical rehabilitation. 2012;26(10):945-51.
66. Hill JC, Kang S, Benedetto E, Myers H, Blackburn S, Smith S, et al. Development and initial cohort validation of the Arthritis Research UK Musculoskeletal Health Questionnaire (MSK-HQ) for use across musculoskeletal care pathways. BMJ Open. 2016;6(8).
67. Hill J, Kang S, Benedetto E, Myers H, Blackburn S, Smith S, et al. PMS74 - Development of the Musculoskeletal Health Questionnaire (MSK-HQ) for Use in Different Conditions and Different Healthcare Pathways. Value in Health. 2016;19(7):A544.
68. Gill S, McBurney H. Reliability of performance-based measures in people awaiting joint replacement surgery of the hip or knee. Physiotherapy research international : the journal for researchers and clinicians in physical therapy. 2008;13(3):141-52.
69. Jones CJ, Rikli RE, Beam WC. A 30-s chair-stand test as a measure of lower body strength in community-residing older adults. Research quarterly for exercise and sport. 1999;70(2):113-9.
70. Bennell K, Dobson F, Hinman R. Measures of physical performance assessments: Self-Paced Walk Test (SPWT), Stair Climb Test (SCT), Six-Minute Walk Test (6MWT), Chair Stand Test (CST), Timed Up & Go (TUG), Sock Test, Lift and Carry Test (LCT), and Car Task. Arthritis Care & Research. 2011;63(S11):S350-S70.

71. French HP, Fitzpatrick M, FitzGerald O. Responsiveness of physical function outcomes following physiotherapy intervention for osteoarthritis of the knee: an outcome comparison study. Physiotherapy. 2011;97(4):302-8.
72. Halket A, Stratford PW, Kennedy DM, Woodhouse LJ. Using hierarchical linear modeling to explore predictors of pain after total hip and knee arthroplasty as a consequence of osteoarthritis. The Journal of arthroplasty. 2010;25(2):254-62.
73. Kennedy DM, Hanna SE, Stratford PW, Wessel J, Gollish JD. Preoperative function and gender predict pattern of functional recovery after hip and knee arthroplasty. The Journal of arthroplasty. 2006;21(4):559-66.
74. Mizner RL, Petterson SC, Clements KE, Zeni JA, Jr., Irrgang JJ, Snyder-Mackler L. Measuring functional improvement after total knee arthroplasty requires both performance-based and patient-report assessments: a longitudinal analysis of outcomes. The Journal of arthroplasty. 2011;26(5):728-37.
75. Podsiadlo D, Richardson S. The Timed "Up & Go": A Test of Basic Functional Mobility for Frail Elderly Persons. Journal of the American Geriatrics Society. 1991;39(2):142-8.
76. Zeni JA, Jr., Axe MJ, Snyder-Mackler L. Clinical predictors of elective total joint replacement in persons with end-stage knee osteoarthritis. BMC musculoskeletal disorders. 2010;11:86.
77. Parmelee PA, Tighe CA, Dautovich ND. Sleep disturbance in osteoarthritis: linkages with pain, disability, and depressive symptoms. Arthritis Care Res (Hoboken). 2015;67(3):358-65.
78. Parmelee PA, Tighe CA, Dautovich ND. Sleep Disturbance in Osteoarthritis: Linkages with Pain, Disability and Depressive Symptoms. Arthritis care & research. 2015;67(3):358-65.
79. Power JD, Perruccio AV, Badley EM. Pain as a mediator of sleep problems in arthritis and other chronic conditions. Arthritis Care & Research. 2005;53(6):911-9.
80. Smith MT, Quartana PJ, Okonkwo RM, Nasir A. Mechanisms by which sleep disturbance contributes to osteoarthritis pain: A conceptual model. Current pain and headache reports. 2009;13(6):447-54.
81. Stebbings S, Herbison P, Doyle TCH, Treharne GJ, Highton J. A comparison of fatigue correlates in rheumatoid arthritis and osteoarthritis: disparity in associations with disability, anxiety and sleep disturbance. Rheumatology. 2010;49(2):361-7.
82. Hawker GA, French MR, Waugh EJ, Gignac MAM, Cheung C, Murray BJ. The multidimensionality of sleep quality and its relationship to fatigue in older adults with painful osteoarthritis. Osteoarthritis and Cartilage. 2010;18(11):1365-71.
83. Hopman-Rock M, Odding E, Hofman A, Kraaimaat FW, Bijlsma JW. Physical and psychosocial disability in elderly subjects in relation to pain in the hip and/or knee. J Rheumatol. 1996;23(6):1037-44.
84. Fung MM, Peters K, Redline S, Ziegler MG, Ancoli-Israel S, Barrett-Connor E, et al. DECREASED SLOW WAVE SLEEP INCREASES RISK OF DEVELOPING HYPERTENSION IN ELDERLY MEN. Hypertension. 2011;58(4):596-603.
85. Levendowski DJ, Popovic D, Berka C, Westbrook PR. Retrospective cross-validation of automated sleep staging using electroocular recording in patients with and without sleep disordered breathing. International Archives of Medicine. 2012;5:21-.
86. Mesarwi O, Polak J, Jun J, Polotsky VY. Sleep disorders and the development of insulin resistance and obesity. Endocrinology and metabolism clinics of North America. 2013;42(3):617-34.
87. Ancoli-Israel S, Cooke JR. Prevalence and comorbidity of insomnia and effect on functioning in elderly populations. J Am Geriatr Soc. 2005;53(7 Suppl):S264-71.
88. Cricco M, Simonsick EM, Foley DJ. The impact of insomnia on cognitive functioning in older adults. J Am Geriatr Soc. 2001;49(9):1185-9.

89. Ancoli-Israel S. Sleep and its disorders in aging populations. Sleep Medicine. 2009;10:S7-S11.
90. Senba E. A key to dissect the triad of insomnia, chronic pain, and depression. Neuroscience letters. 2015;589:197-9.
91. Hughes JM, Song Y, Fung CH, Dzierzewski JM, Mitchell MN, Jouldjian S, et al. Measuring Sleep in Vulnerable Older Adults: A Comparison of Subjective and Objective Sleep Measures. Clinical Gerontologist. 2017:1-13.
92. Lundeen J, McCall WV, Krystal A, Looney S. Validating functional data analysis measures from 24-h actigraphy data. Biological Rhythm Research. 2018:1-14.
93. Kushida CA, Chang A, Gadkary C, Guilleminault C, Carrillo O, Dement WC. Comparison of actigraphic, polysomnographic, and subjective assessment of sleep parameters in sleep-disordered patients. Sleep Medicine. 2001;2(5):389-96.
94. Sadeh A, Hauri PJ, Kripke DF, Lavie P. The Role of Actigraphy in the Evaluation of Sleep Disorders. Sleep. 1995;18(4):288-302.
95. Lichstein KL, Stone KC, Donaldson J, Nau SD, Soeffing JP, Murray D, et al. Actigraphy Validation with Insomnia. Sleep. 2006;29(2):232-9.
96. de Souza L, Benedito-Silva AA, Pires MLN, Poyares D, Tufik S, Calil HM. Further Validation of Actigraphy for Sleep Studies. Sleep. 2003;26(1):81-5.
97. Backhaus J, Junghanns K, Broocks A, Riemann D, Hohagen F. Test–retest reliability and validity of the Pittsburgh Sleep Quality Index in primary insomnia. Journal of Psychosomatic Research. 2002;53(3):737-40.
98. Carpenter JS, Andrykowski MA. Psychometric evaluation of the pittsburgh sleep quality index. Journal of Psychosomatic Research. 1998;45(1):5-13.
99. Buysse DJ, Reynolds IIICF, Monk TH, Hoch CC, Yeager AL, Kupfer DJ. Quantification of Subjective Sleep Quality in Healthy Elderly Men and Women Using the Pittsburgh Sleep Quality Index (PSQI). Sleep. 1991;14(4):331-8.
100. Finan PH, Buenaver LF, Bounds SC, Hussain S, Park RJ, Haque UJ, et al. Discordance between pain and radiographic severity in knee osteoarthritis: Findings from quantitative sensory testing of central sensitization. Arthritis & Rheumatism. 2013;65(2):363-72.
101. Taibi DM, Vitiello MV. A pilot study of gentle yoga for sleep disturbance in women with osteoarthritis. Sleep Medicine. 2011;12(5):512-7.
102. Palmieri-Smith RM, Thomas AC, Karvonen-Gutierrez C, Sowers MF. Isometric Quadriceps Strength in Women with Mild, Moderate, and Severe Knee Osteoarthritis. American journal of physical medicine & rehabilitation / Association of Academic Physiatrists. 2010;89(7):541-8.
103. Tan J, Balci N, Sepici V, Gener FA. Isokinetic and isometric strength in osteoarthrosis of the knee. A comparative study with healthy women. Am J Phys Med Rehabil. 1995;74(5):364-9.
104. Berry PA, Jones SW, Cicuttini FM, Wluka AE, Maciewicz RA. Temporal relationship between serum adipokines, biomarkers of bone and cartilage turnover, and cartilage volume loss in a population with clinical knee osteoarthritis. Arthritis & Rheumatism. 2011;63(3):700-7.
105. Richette P, Poitou C, Garnero P, Vicaut E, Bouillot J-L, Lacorte J-M, et al. Benefits of massive weight loss on symptoms, systemic inflammation and cartilage turnover in obese patients with knee osteoarthritis. Annals of the Rheumatic Diseases. 2011;70(1):139-44.
106. Fransen M, McConnell S, Harmer AR, Van der Esch M, Simic M, Bennell KL. Exercise for osteoarthritis of the knee: a Cochrane systematic review. British Journal of Sports Medicine. 2015;49(24):1554-7.
107. Moseley JB, O'Malley K, Petersen NJ, Menke TJ, Brody BA, Kuykendall DH, et al. A controlled trial of arthroscopic surgery for osteoarthritis of the knee. The New England journal of medicine. 2002;347(2):81-8.

108. Rosenberg TD, Paulos LE, Parker RD, Coward DB, Scott SM. The forty-five-degree posteroanterior flexion weight-bearing radiograph of the knee. The Journal of bone and joint surgery American volume. 1988;70(10):1479-83.
109. Kellgren JH, Lawrence JS. Radiological Assessment of Osteo-Arthrosis. Annals of the Rheumatic Diseases. 1957;16(4):494-502.
110. Kohn MD, Sassoon AA, Fernando ND. Classifications in Brief: Kellgren-Lawrence Classification of Osteoarthritis. Clinical Orthopaedics and Related Research. 2016;474(8):1886-93.
111. Foley A, Halbert J, Hewitt T, Crotty M. Does hydrotherapy improve strength and physical function in patients with osteoarthritis—a randomised controlled trial comparing a gym based and a hydrotherapy based strengthening programme. Annals of the Rheumatic Diseases. 2003;62(12):1162-7.

B. Statistical Analysis Plan

Section 1: Administrative Information

Title:
Statistical analysis plan SAP for a randomised controlled trial evaluating the efficacy of Internet-based Exercises Aimed at Treating Knee Osteoarthritis (iBEAT-OA)

Trial Registration number:
NCT03545048

SAP version:
V1

Protocol Version:
V1 published and link:
https://bmjopen.bmj.com/content/9/10/e030564

SAP Revisions:
Not applicable

Roles and Responsibilities:
Sameer Gohir – writing SAP in collaboration with other co-authors
Phd Scholar
NIHR Nottingham Biomedical Research Centre, School of Medicine, University of Nottingham, Nottingham, East Midland, UK

Frida Eek – writing SAP in collaboration with other co-authors
Associate Professor, RPT
Department of Health Sciences
Lund University

Ana M. Valdes – SAP writer and Chief Investigator
NIHR Nottingham Biomedical Research Centre, School of Medicine, University of Nottingham, Nottingham, East Midland, UK

Section 2: Introduction

Background

Osteoarthritis is the most common cause of disability in the elderly population and most individuals suffering from osteoarthritis is managed in the primary care setting (1). Knee osteoarthritis is the most common form of arthritis in the world (2). The rate of knee arthritis is as high as that of cardiac disease and is the most common problem in the individuals over the age of 65 (3). In the United Kingdom, 10% of individuals between the ages of 65 and 74 consult their general practitioners about osteoarthritis every year (4). 4% of general population see their general practitioners as a result of knee osteoarthritis, and half of them (2%) consult their general practitioner for the first time or with an acute flare of knee arthritis (1).
According to national and international guidelines, the first line of treatment for osteoarthritis pain (OA) is non-surgical; exercise, information and, in relevant cases, weight loss (5-9). There is sufficient evidence for the effectiveness of exercises in the management of knee osteoarthritis and to improve the functional capacity of these individuals to cope better with the activities of daily living (10-24). In fact, one study reported a slow-down of progressive radiographic changes of knee OA as a result of

strengthening exercises (25). There is a significant disparity on the effectiveness of different types of exercises for the knee osteoarthritis, and a combination of open and closed isotonic exercises are recommended knee osteoarthritis (26) with the exception to those individuals who find these exercises difficult and painful. In such groups, isometric exercises of knee muscles should be considered (26). In an attempt to manage the knee osteoarthritis, one can exacerbate the symptoms of knee arthritis by following ineffective or unsafe exercises leading to poor prognosis and poor adherence to these exercises (27), hence the need to choose the exercises carefully.

There are no standardised exercise programs for knee OA in the United Kingdom. Arthritis Research UK (ARUK) recommend knee exercises for knee pain which is a generic term and covers soft tissue injuries (28). These exercises are normally recommended by the general practitioners as the first line of protocol when they consult someone with the knee pain. If these exercises fail to make remarkable progress, individuals with knee OA are referred to the Physiotherapy department. As there is no standardised protocol, therefore various exercises are given by the physiotherapists which vary in term of contents, intensity and duration. These exercises may help some individuals and may not help others. One example is that a patient may get recommended generic knee exercises which may help individuals with knee OA, however, may flare up the pain in the individuals with the patellofemoral joint syndrome or vice versa. Therefore, there is a need for standardised exercises to address knee OA pain which can ideally be accessed online. The exercise programme which we will use is a part of the web-based exercises which is derived from the Supported Osteoarthritis Self-Management Programme (SOASP) developed in Sweden. In the SOASP, scientific evidence for information and exercise in conjunction with osteoarthritis was put into clinical practice. From 2008 until January 2017, around 75,000 patients had participated the SOASP, 2339 physiotherapists and occupational therapists were educated to hold the Supported Osteoarthritis Self-Management Programme, and today SOASP is offered in 700 units all over Sweden (https://boa.registercentrum.se/boa-in-english/better-management-of-patients-with-osteoarthritis-boa/p/By_o8GxVg). This intervention has been rated as good or as very good by 94% of patients and has an excellent safety profile and acceptability (29).

Because of the high prevalence of knee arthritis, strategies to deliver exercise interventions that are both efficacious and cost-effective becomes a priority. Some of the issues relating to the delivery of exercise interventions for knee OA include compliance, accessibility to clinics for people with mobility problems and cost of delivery of such services. There are previous studies which have assessed the efficacy of home-based exercises and reported good results (12, 14, 30) however very few studies looked at a web-based delivery of exercises on the knee arthritis (31-35). Unfortunately, most of these studies recruited patients with knee pain, and radiographic evidence of knee osteoarthritis was only assessed in a single study (33). Some of these exercises can be used for non-specific knee pain including cases of soft tissue injuries, arthritic knees, patellofemoral syndrome or any other reason for knee pain. However, generalising these results on the group of patients with knee osteoarthritis warrants a risk of increasing their pain just in case that those exercises are strenuous or more demanding. Therefore, there is a need for an exercise programme which is specific for osteoarthritis and economical so individuals can access them in their own homes.

Most individuals lose interest in exercising regularly after they finish with their physiotherapy intervention. There are various reasons for this including adjusting lifestyle to include these exercises, pain during the exercises, lack of motivation and lack of professional to monitor the progress (36-38). So, there is a need for the

intervention which keeps them motivated and improves the compliance and that actually encourages forming a habit of regular exercising.
Knee OA pain is accompanied by a number of additional disturbances that influence the individual's health which we also propose to study in conjunction with pain relief due to exercise. There is well known link between sleep disturbances and chronic pain and both epidemiological studies (39-41) and experimental studies (42-46) have established a link between disturbed sleep and knee OA. These studies confirmed that individuals with OA have the higher suffering from sleep disturbances. Sleep disturbances have been recognised as an important factor in determining the perception of pain in individuals (46). A relationship between sleep disturbances and pain severity in knee OA patients is usually explored, and sleep disturbances such as shortened sleep duration and fragmented sleep (39) have been associated with increased sensitivity to pain in OA patients and consequently with decreased quality of life (41). Therefore, studying the sleeping pattern is highly relevant if we are discussing a successful online exercise programme for knee OA.
Recent epidemiological and clinical studies have also underlined that metabolic syndrome (MetS) has the most significant impact on the initiation and severity of OA (47-51). Metabolic triggered inflammation, also known as meta-inflammation (52) can be a result of abnormalities in body composition, adipokines, cytokines, lipids and vitamin D and has been associated with the pathogenesis of OA (50). However, the intricate links between exercises, sleep, pain, metabolic syndrome and OA are not fully understood. To date, the relationship between improvements in pain due to exercise for pain relief and other changes related to health parameters that have been linked to OA or chronic pain has not been explored. Specifically, the effects of exercise for knee pain relief on sleep, biomarkers of inflammation and insulin resistance is unknown. We aim to study these parameters and establish the link between exercises for knee OA and these parameters.

Rational
This study aims to explore the benefits of internet-based exercises in the patients with knee osteoarthritis to establish if their pain decreases after six weeks. Being a web-based set of activities, it makes it more accessible to the patients living in rural areas or who have difficulty assessing a clinic and should make it more cost-effective and by cutting down the traveling time and freeing up the time of the physiotherapists. Another reason is to establish if we can have a standardised intervention for knee OA which keeps the individuals motivated, and they can manage their pain. Lastly, we will endeavour to explore the complicated relationship between chronic pain, sleep, biomarkers of inflammation and knee osteoarthritis. Our study is different from the other studies as there is not a single study (to our knowledge) which has studied web-based intervention on United Kingdom population of knee osteoarthritis. Furthermore, we will endeavour to recruit only those individuals who have radiographic evidence of knee osteoarthritis. The purpose of these inclusion criteria is to rule out other causes of knee pain such as soft tissue injuries or patellofemoral syndrome and to establish the efficacy of this programme specifically in individuals with knee OA. Therefore, this study can help in generation of recommendations for the clinical setups where most of the symptomatic patients have radiographic evidence of knee osteoarthritis.

Objectives

Primary Objective

The primary aim of the study is to test the effect of an internet-based exercise treatment program on pain, measured on a Numerical Rating Scale (NRS), in knee OA.

Secondary Objectives

Secondary aims of the study are to test the effect of an internet-based exercise treatment program on the following outcomes:

1. Sleep
2. Pain (Pressure pain threshold – PPT, Temporal summation – TS, and conditional pain modulation – CPM)
3. Inflammatory markers on ultrasound (Synovial fluid, synovial hypertrophy and hyper vascularity) (MSK-USS)
4. Muscle Thickness Assessment (MTA) of Vastus Lateralis Oblique (VLO) using MSK-USS
5. Maximum Voluntary Contraction (MVC)- isokinetic contraction of Quadriceps muscle
6. Biomarkers of insulin resistance
7. Physical functioning (Timed up and go test, 30-second sit to stand test)
8. Western Ontario and McMaster Universities Osteoarthritis Index (WOMAC)
9. Pittsburgh Sleep Quality Index (PSQI)
10. General health questionnaire (MSK- HQ)

Section 3: Study Methods

Trial Design

The iBEAT-OA study is a randomised controlled trial in the primary care setting at Nottingham with participants suffering from knee osteoarthritis, 1:1 randomised to web-based exercises or ‘usual care’. The study has been approved by the sponsor, Research Ethical committee (REC) and Health Research Authority (HRA).

Randomisation

Participants will undergo computer-generated stratified randomisation by a statistician at Academic Rheumatology using the https://sealedenvelope.com/ software. Generation of a randomisation schedule will include obtaining the random numbers and assigning random numbers to each subject under the specific treatment conditions. Participants will be equally allocated between treatment arms with at least n=67 per arm. As the online system will be used for randomisation, therefore, code break will be kept online, and the research manager will be responsible for keeping a track of it.

As per cohort multiple randomised controlled trial design mentioned by another study (54), individuals will have an assessment to establish their suitability for this study and once that has been established, we will randomise them into control group and interventional group. At this occasion, individuals will find out that they belong to one group or the other and hence blinding is not possible. Because the lack of masking might affect the motivation of participants randomised into the control arm, individuals in the control arm will be offered access to the web-based exercise programme after the six weeks of the study, if they wish to use it to avoid a nocebo effect in this group. Blinding such interventional studies are difficult to achieve as participants will establish as to in which group they are in, however, the examiner will be blinded to the intervention.

Sample size
The sample size calculation is based on a standardised effect size of 0.49, corresponding to a group difference in primary outcome VAS pain of 12 points on a scale of 0-100 (corresponding to a mean difference of 1,2 points on a 1-10 NRS scale). This difference was found in a recent systematic review of 44 high-quality exercise trials for knee OA pain (3537 participants) (56). With this estimated effect, 60 participants in each group is required to achieve 75% power to reject the null hypothesis, with alpha level set to 0.05.

Framework
We hypothesise that i-BEAT OA is superior to “usual care” in primary care setup, in order to decrease pain reported by OA patients.

Statistical interim Analyses and stopping guidance
No interim analysis was planned or performed. As there was no adverse event expected, the trial was not planned to be stopped early.

Timing of Final Analysis
The final analyses will be performed once the trial has stopped and all follow up data has been collected.

Timing of outcome assessments
All outcome variables were collected at baseline, and at follow-up (6 weeks post baseline) in both arms. However, trial was stopped on 19th March 2020 due to the COVID-19 pandemic resulting in having only 105 eligible participants completing both visits.

Section 4: Statistical Principles

Confidence intervals and p-values
Alpha level will be set to 0.05. 95% confidence intervals will be presented (if data allows for parametric analyses).

Adjustments for multiplicity will not be performed. However, primary outcome is defined and will be the main focus for the results interpretation. Remaining analyses will be considered exploratory, and interpreted in light of the general pattern of effects rather than solely based on individual p-values.

Adherence to the i-BEAT OA will be defined as percentage patient completed activities of those distributed by the platform per week. Number of non-compliant participants will be reported.

Analyses will be based on the Intention to treat (ITT) population. Since there are no risks associated with i-BEAT OA, safety analyses will not be performed.

Section 5: Trial Population

Screening Data
A selection of eligible people for the study will be invited from existing databases held at Academic Rheumatology, City Hospital Nottingham of participants with knee pain who have agreed to be contacted for future studies. Any shortfall in the recruitment will be compensated by sending study leaflet to GP surgeries.

Eligibility
Potentially eligible people will be all men and women, aged between 45 years and older, registered with the GP surgery. The screening of existing records can be done by members of their direct healthcare team for those who had x-rays and osteoarthritis of the knee had been established. The GPs will follow the inclusion and exclusion criteria.

INCLUSION CRITERIA

- Aged 45 years and onward
- Clinical diagnosis of knee arthritis with complaints of knee pain for 3-6 months, early morning stiffness <30 minutes, crepitus, bony tenderness, and no palpable warmth and radiographically established osteoarthritis (at least score 1 on K/L scale)
- Able to read and write English
- Able to use/access computer or tablet and have access to the internet

EXCLUSION CRITERIA

- Inability to give informed consent
- Terminal or mental illness
- Neurological conditions (Stroke, Multiple Sclerosis, Parkinson’s, Motor Neuron Disease, Muscular Dystrophy, Huntington’s disease), inflammatory joint diseases including rheumatoid arthritis, gout or calcium pyrophosphate deposition disease (CPPD), and dementia
- Participants with sleep apnea previously diagnosed by a physician
- Acute soft tissue injury to the knee within last 3 months before recruiting diagnosed by a physician
- Unstable heart condition or rapid fluctuations in hypertension previously diagnosed by a physician

Recruitment
Recruitment will be presented as CONSORT flow diagram.

Withdrawal / Follow-up

This will be presented in the article as per CONSORT guidelines. Loss to follow up will be presented as proportion of participants in each group not responding to the 6 week follow up survey.

Baseline patient characteristics

Descriptive baseline characteristics regarding age, sex, Body Mass Index (BMI) and Kellgren-Lawrence (KL) score will be presented for each group.

Sex will be presented as proportions (%). Age and BMI will be presented as mean (SD). KL score will be presented as median, mean (SD), and proportion in each category.

Section 6: Analysis

Outcome variables

All outcome variables will be measured at baseline, and at follow up (after 6 weeks). The difference between baseline and follow up will be calculated and used as outcome measure in the analyses/group comparisons.

Primary outcome:
NRS pain (0-10)

Secondary outcomes:

1. Sleep Quality: This was assessed by using actigraphy device known as ActTrust (53). A single device was given to each participant during baselines assessment session and was collected back during the follow-up session.
2. Pain sensitivity (Pressure pain thresholds – PPT, Temporal summation – TS, and conditional pain modulation – CPM).
3. Inflammatory markers on ultrasound (Synovial fluid, synovial hypertrophy and hyper vascularity) (MSK-USS)
4. Muscle Thickness Assessment (MTA) of Vastus Lateralis Oblique (VLO) using MSK-USS. This was dropped from the study due to lack of Panoramic view on Toshiba Aplio SSA-770A machine as the machine did not allow to find the mid-portion of the VLO to have valid and repeatable measurements.
5. Maximum Voluntary Contraction (MVC)- isokinetic contraction of Quadriceps and hamstring muscles using Computer Sports Medicine, Inc (CSMI) HUMAC / NORM Testing and Rehabilitation System (Model 770).
6. Physical functioning (Time up and go test, 30-second sit to stand test)
7. Western Ontario and McMaster Universities Osteoarthritis Index (WOMAC)
8. Pittsburgh Sleep Quality Index (PSQI)
9. General health questionnaire (MSK- HQ)

Analysis methods

The distribution for each difference score will be evaluated. If the score follows an approximately normal distribution with no apparent outliers, between group differences will be analysed through ANOVA with adjustment for baseline scores (ANCOVA). Due to the randomisation, no systematic differences between groups are expected and hence no control for further "confounding" will be performed. Results for the between group comparison will be presented as "difference in mean difference" with accompanying 95% confidence intervals.

Within group differences from baseline to follow up will be evaluated through pre-post mean difference with accompanying 95% CI. Differences/changes with confidence intervals not covering "0" will be considered statistically significant. Standardised effect sizes (Cohen's D) will be computed (mean difference/baseline SD).

If the difference scores clearly deviate from a normal/symmetric distribution, the comparisons will instead be performed through non-parametric analyses. For the

between group comparisons, Mann-Whitney test will be applied and for the within group comparison, Wilcoxon signed rank test will be used. Results will be presented as median scores (IQR) and p-values. No adjustment for baseline scores will be performed, as the non-parametric tests do not allow for this.

Missing data
Missing data will be handled by group mean imputation.

Harm
No risks are associated with the intervention; however we will record any serious adverse event or harms and will report in publications.

Statistical software
The data will be analysed using StatsDirect V3.2.10, SigmaXL 8.15 and SPSS v24
