# Supplementary material for: Effectiveness of Internet-Based Exercises Aimed at Treating Knee Osteoarthritis: The iBEAT-OA Randomized Clinical Trial
Source: JAMA Netw Open. 2021 Feb 23;4(2):e210012. doi: 10.1001/jamanetworkopen.2021.0012 (PMC7903254; doi:10.1001/jamanetworkopen.2021.0012)
Supplement: Supplement 3. — Data Sharing Statement [file jamanetwopen-e210012-s003.pdf]

Data Sharing Statement

Gohir. Effectiveness of Internet-based Exercises Aimed at Treating Knee Osteoarthritis. *JAMA Netw Open*. Published February 23, 2021. doi:10.1001/jamanetworkopen.2021.0012

Data
Data available: Yes
Data types: Deidentified participant data
How to access data: The request for any data should be sent to Sameer Gohir on sameer.gohir@nottingham.ac.uk
When available: With publication

Supporting Documents
Document types: None

Additional Information
Who can access the data: The data can be shared with researchers.
Types of analyses: Syetematic Review / Meta-Analysis
Mechanisms of data availability: With investigator support and would require approval from the sponsor along with signed data access agreement.
